# TransBic: bucket trend-preserving biclustering for finding local and interpretable expression patterns

**DOI:** 10.1093/bib/bbaf050

**Published:** 2025-02-05

**Authors:** Jing Li, Qinglin Mei, Chaoxia Yang, Naibo Zhu, Guojun Li

**Affiliations:** Research Center for Mathematics and Interdisciplinary Sciences, Shandong University, No. 72 Binhai Road, Jimo Distinct, Qingdao 266237, Shandong, China; Institute of Systems Engineering, PLA Academy of Military Sciences, No. 28 Xizhimen North Street, Haidian Distinct, Beijing 100082, China; MOE key Laboratory of Bioinformatics, BNRIST Bioinformatics Division, Department of Automation, Tsinghua University, No. 30 Shuangqing Road, Beijing 100084, China; College of Science, Nanjing University of Posts and Telecommunications, No. 9 Wenyuan Road, Yadong New City District, Nanjing 210023, Jiangsu, China; Institute of Systems Engineering, PLA Academy of Military Sciences, No. 28 Xizhimen North Street, Haidian Distinct, Beijing 100082, China; Research Center for Mathematics and Interdisciplinary Sciences, Shandong University, No. 72 Binhai Road, Jimo Distinct, Qingdao 266237, Shandong, China

**Keywords:** biclustering, local pattern identification, acyclic tournament digraph

## Abstract

Biclustering has emerged as a promising approach for analyzing high-dimensional expression data, offering unique advantages in uncovering localized co-expression patterns that traditional clustering methods often miss and thus facilitating advancements in complex disease research and other biomedical applications. However, state-of-the-art methods identify distinct patterns at the expense of losing information about specific patterns, some of which have been used to define cancer subtypes or reflect the progression of a disease or cellular processes. Additionally, these methods exhibit poor effectiveness in noisy environments. To address these limitations, we propose the bucket trend-preserving (BTP) pattern, a novel generalization of existing patterns. And we have developed an algorithm, TransBic, to extract significant biclusters of BTP-patterns. Specifically, TransBic transforms the problem into identifying common multipartite acyclic tournament subdigraphs shared by distinct subsets of acyclic tournament digraphs derived from a given expression matrix. Compared with prominent tools, TransBic demonstrates superior performance in identifying biclusters of all non-row-constant patterns, especially under noise and data fluctuations. Furthermore, TransBic successfully identifies the most disease-related pathways for type 2 diabetes (T2D), colorectal cancer, hepatocellular carcinoma, and breast cancer, outperforming other tools in this regard. Different from previous generalizations, BTP-patterns capture specific up-regulation and down-regulation dynamics. Through targeted analysis of BTP-patterns in T2D expression data, TransBic uncovers biological processes affected by disease risk factors, extending the application of trend-preserving biclustering in expression data analysis.

## Graphical Abstract

**Graphical Abstract:**
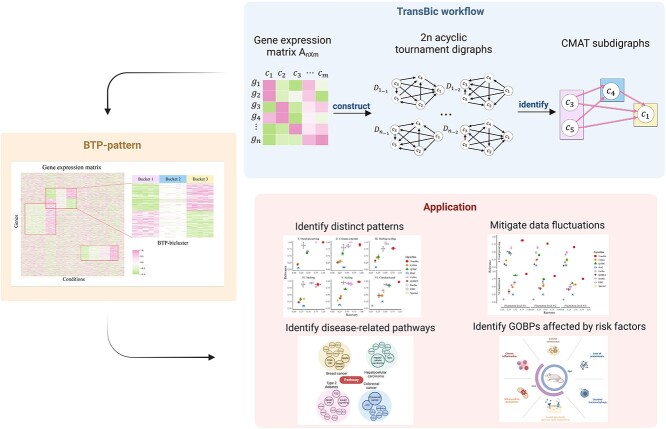

## Introduction

Gene expression data, characterized by high dimensionality and heterogeneity, often result in localized co-expression patterns that reveal intricate relationships between certain genes and conditions. Unlike conventional approaches that cluster genes or conditions independently, biclustering simultaneously identifies meaningful associations between subsets of genes and conditions, facilitating the discovery of these local patterns [1]. To date, numerous significant local patterns have been proposed, including constant [2], shifting, scaling, shifting-scaling [3], checkerboard [4], order-preserving [5], and trend-preserving [6]. These patterns characterize functional gene modules (FGMs) from distinct perspectives: similar expression levels, consistent translational changes, proportional changes in expression, and coordinated up- or down-regulation across different conditions. In addition, the consistent expression changes of FGMs implied by these patterns have garnered considerable attention in precision medicine, disease stratification, and understanding disease progression [5, 7, 8]. Comprehensive pattern identification is pivotal in analyzing expression data, as it extracts a richer tapestry of biologically significant information compared with the limited insights garnered from isolated patterns. However, most biclustering algorithms find only one or a few of these patterns [1]. Wang *et al*. [6] proposed a generalization of trend-preserving patterns, encompassing all the above patterns except the checkerboard as specific instances. For a bicluster with the generalization, the values in each row (gene) are monotonically increasing, not necessarily strict, along a common permutation of columns (conditions) in a expression matrix. The current biclustering algorithms for identifying distinct patterns, including UniBic [6], EBIC [9], RecBic [10], and ARBic [11], are all based on identifying such a generalized pattern. Specifically, each bicluster is explored by iteratively adding rows while removing columns that do not conform to the pattern, or by adding columns while removing non-conforming rows. While these algorithms have achieved great success, they exhibit two prominent limitations.

Firstly, they fail to retain specific expression changes for identified patterns. These algorithms output all biclusters based on the generalized trend-preserving pattern, without figuring out the conditions under which gene expression levels remain stable or are coordinately up-regulated or down-regulated. Consequently, although these algorithms can identify FGMs characterized by various patterns, they fail to emphasize their common expression changes. Nevertheless, these common changes provide valuable insights for biological studies, including cancer subtyping and reflecting the progression of biological processes. Additionally, these algorithms would incorporate genes that fail to be consistently up-regulated or down-regulated into the same bicluster (see an example in Fig. S1).

Secondly, they still lack robustness against noise and data fluctuations. Noise and data fluctuations will disrupt the true ranking of gene expression values. UniBic, RecBic, and ARBic adopt the approach developed by Li *et al*. [12] to convert the expression values of each gene into a set of consecutive ranks. The user determines a custom parameter—the number of ranks, typically fewer than the number of conditions—where each rank corresponds to an equal number of expression values, and then performs biclustering on the resulting ranking matrix. While this approach can smooth out minor fluctuations, the pre-set ranks inevitably introduce noise due to misclassifications of expression values, thereby affecting the accuracy of subsequent biclustering.

**Figure 1:**
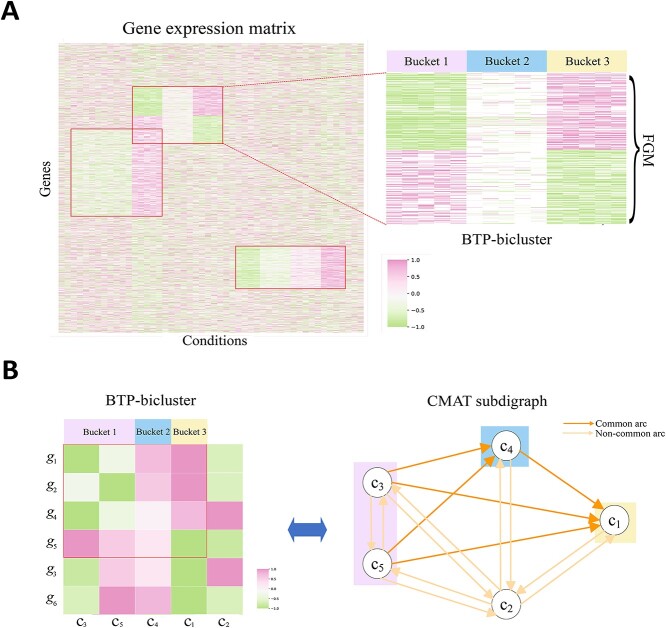
Overview of TransBic. A. In a gene expression matrix, a BTP-bicluster is composed of a subset of rows (genes) and columns (conditions), where genes exhibit a BTP-pattern under the conditions. Specifically, either genes have their expression values up-regulated from Bucket 1 to Bucket 2, and then to Bucket 3, or down-regulated in the same sequential manner. For the BTP-bicluster, its all genes constitute an FGM. B. For the BTP-bicluster containing genes $g_{1},g_{2},g_{4},\ and\ g_{5}$ and conditions $c_{3},c_{5},c_{4},\ and\ c_{1}$, the genes exhibit a BTP-pattern in this partition $\{c_{3},c_{5}\},\{c_{4}\},\{c_{1}\}$: $\max \{a_{i,j}: c_{j} \in \{c_{3}, c_{5}\}\} < \min \{a_{i,j}: c_{j} \in \{c_{4}\}\}$, $\max \{a_{i,j}: c_{j} \in \{c_{4}\}\} < \min \{a_{i,j}: c_{j} \in \{c_{1}\}\}$ for $i = 1, 2, 4$, and $\min \{a_{i,j}: c_{j} \in \{c_{3}, c_{5}\}\}> \max \{a_{i,j}: c_{j} \in \{c_{4}\}\}$, $\min \{a_{i,j}: c_{j} \in \{c_{4}\}\}> \max \{a_{i,j}: c_{j} \in \{c_{1}\}\}$ for $i = 5$. Correspondingly, the acyclic tournament digraphs $D_{1_{-1}},D_{2_{-1}},D_{4_{-1}},\ and\ D_{5_{-2}}$ share a CMAT subdigraph with partite sets $\{c_{3},c_{5}\},\{c_{4}\}$, and $\{c_{1}\}$. In the CMAT subdigraph, each partite set has no internal arcs, and for any two vertices from different partite sets, there exists an arc. The direction of these arcs is as follows: from $c_{3},c_{5}$ to $c_{4}$, and from $c_{3},c_{5},c_{4}$ to $c_{1}$.

To overcome these limitations, we propose a new generalization of trend-preserving patterns. The generalized pattern relaxes each of the conditions under which genes exhibit a consistent expression trend into a subset of conditions, called a bucket. Accordingly, the generalized pattern is termed a bucket trend-preserving (BTP) pattern. A comparable relaxation approach for order-preserving patterns has previously been introduced [13]. The constant pattern can be classified into row-constant and column-constant, with the BTP-pattern serving as a generalization of all non-row-constant patterns. This indicates that TransBic is especially well suited for analyzing expression data with differential expression of interest. For a bicluster of BTP-patterns, all buckets could be arranged in such a way that for each gene, the expression values under each bucket are strictly higher than those under the next bucket, or vice versa. These buckets constitute a partition of all conditions in the bicluster. We say that the bicluster is of BTP-patterns in this partition. For convenience, we refer to the bi-cluster as BTP-bicluster. For a BTP-bicluster, genes in the same bucket exhibit stable expression levels, while genes across different buckets can be coordinately up-regulated, down-regulated, or a combination of both: some genes are up-regulated while others are down-regulated (see an example in Fig. 1A). Analogous to the ranks mentioned above, these buckets help smooth out fluctuations. However, they are not intended for individual genes but rather provide a robust and accurate representation of common expression trends. These buckets are not predefined, avoiding the introduction of errors as mentioned above. Instead, they are generated automatically during the bicluster search process of our proposed algorithm, TransBic. This flexibility enables TransBic to recognize diverse expression patterns more effectively. Inspired by the identification of BTP-biclusters, we have developed a novel algorithm, TransBic. Constrained by column operations in the expression matrix, TransBic transforms the problem into the detection of common multipartite acyclic tournament (CMAT) subdigraphs by introducing a specific graph structure: the acyclic tournament digraph [14].

We evaluated TransBic against 10 other state-of-the-art tools, QUBIC [12], UniBic [6], QUBIC2 [15], EBIC [9], RecBic [10], FABIA [16], Spectral [4], ISA [17], RUBic [18], and MESBC [19], using synthetic datasets. TransBic consistently outperformed them in identifying BTP-biclusters (refer to the tool selection in the Supplementary Note). In addition, we compared all tools in detecting biclusters of distinct non-row-constant patterns, and TransBic demonstrated superior overall performance. When data fluctuations were introduced, TransBic improved accuracy by at least 23% over other tools. Moreover, we selected six real expression datasets from four distinct diseases: type 2 diabetes, colorectal cancer, hepatocellular carcinoma, and breast cancer, for comparison. We selected pathways specifically related to these diseases as the benchmark. Experimental results indicate that TransBic surpasses 13 state-of-the-art tools, including the above 10 tools as well as DESMOND [7], BiCoN [8], and MoSBi [20], as it identifies the most Kyoto Encyclopedia of Genes and Genomes (KEGG) [21] pathways related to corresponding diseases. TransBic also demonstrated a unique ability to capture specific up- and down-regulation patterns in identified FGMs. Building on this capability, we used TransBic to identify biological processes influenced by disease risk factors in T2D expression datasets. The underlying principle is that these risk factors regulate the expression of key FGMs, which are expected to be enriched in relevant gene ontology biological process (GOBP) [22] terms. Our findings closely align with major conclusions from previous studies, further validating TransBic’s effectiveness.

## Materials and methods

Gene expression profiles are commonly represented by an expression matrix $\mathbf{A}_{n\times m}$, where the $n$ rows correspond to gene $g_{1}$, $g_{2}$, $\dots $, $g_{n}$, and the $m$ columns correspond to conditions $c_{1}$,$c_{2}$, $\dots $, $c_{m}$. Each entry $a_{i,j}$ in the matrix $\mathbf{A}$ represents the expression value of the $i$th gene $g_{i}$ under the $j$th condition $c_{j}$. The BTP-pattern generalizes distinct patterns, enabling the identification of FGMs while capturing their specific up- and down-regulation patterns. Thus, we aim to identify all BTP-biclusters from this expression matrix. However, identifying BTP-patterns in the matrix requires removing non-conforming columns and defining buckets, which imposes a significant burden on column operations. TransBic reformulates the problem as a graph-based problem by introducing the acyclic tournament digraph (see Step 1 for details). Thus, genes exhibit a local BTP-pattern, and their corresponding acyclic tournament digraphs share a CMAT subdigraph, with partite sets representing all buckets (as illustrated in Fig. 1B). Therefore, the problem of identifying BTP-biclusters is transformed into detecting significant CMAT subdigraphs from 2$n$ acyclic tournament digraphs derived from the gene expression matrix.

**Figure 2:**
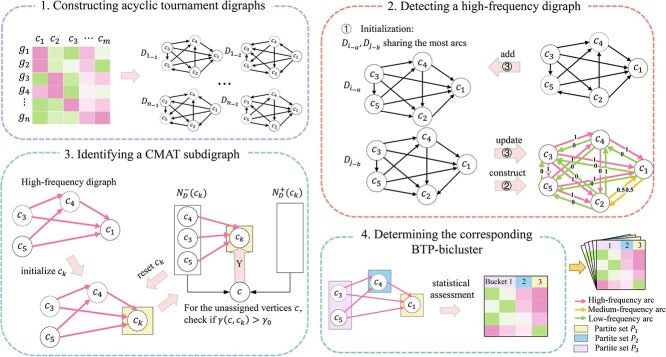
Flowchart of TransBic.

Consider the significant CMAT subdigraph, which is shared by set $S$ of acyclic tournament digraphs. Inspired by other trend-preserving biclustering algorithms, we identify the CMAT subdigraph by iteratively refining $S$, updating the set $H$ to include arcs shared by all digraphs in $S$, until $H$ stabilizes. All the arcs of this CMAT subdigraph are contained in $ H$ according to the definition of $H$. To account for noise in real expression datasets, $H$ is relaxed to include arcs that occur with high frequency in $S$. We then construct the digraph $D=(V,A)$, where $V={c_{1},c_{2},\dots ,c_{m}} $ and $A=H$. The CMAT subdigraph corresponds to a subgraph of $D$ that exhibits a multipartite, acyclic tournament structure. For convenience, we call the digraph $D$ high-frequency digraph (see Fig. 2 for a flowchart of TransBic).

### The TransBic algorithms

**Step 1. Constructing acyclic tournament digraphs.** Given gene expression matrix $\mathbf{A}_{n\times m}$, we construct 2$n$ acyclic tournament digraphs for $n$ genes as follows. For gene $g_{i}$, $i=1,2,\dots ,n$, one digraph is defined as 


(1) \begin{align*}& D_{i_{-1}} \kern-0.3em =\kern-0.3em (V,A_{i_{-1}}), V \kern-0.3em =\kern-0.3em \{c_{1}, c_{2}, \dots, c_{m}\}, (c_{p}, c_{q}) \kern-0.3em \in \kern-0.3em A_{i_{-1}} \kern-0.2em \Leftrightarrow \kern-0.2em a_{i,p} \kern-0.3em < \kern-0.3em a_{i,q}\end{align*}

and the other as 


(2) \begin{align*}& D_{i_{-2}} \kern-0.3em =\kern-0.3em (V,A_{i_{-2}}), V \kern-0.3em =\kern-0.3em \{c_{1}, c_{2}, \dots, c_{m}\}, (c_{p}, c_{q}) \kern-0.3em \in \kern-0.3em A_{i_{-2}} \kern-0.2em \Leftrightarrow \kern-0.2em a_{i,p}\kern-0.3em> \kern-0.3em a_{i,q}\end{align*}

**Step 2. Detecting a high-frequency digraph.** To identify the high-frequency digraph $D$, we only need to determine its arcs $H$. To this end, we initialize $S=\{D_{i_{-a}}, D_{j_{-b}}\}$, where $i,j\in \{1,2,\dots ,n\}$,$i\neq j$ and $a, b\in \{1,2\}$. $D_{i_{-a}}\ and\ D_{j_{-b}}$ represent the pair of digraphs that share the most arcs among $2n$ acyclic tournament digraphs. Let $\overleftrightarrow{D}$ be the complete digraph defined on $V=\{c_{1},c_{2},\dots ,c_{m}\}$. $A(\overleftrightarrow{D})=\{(c_{p}, c_{q}),(c_{q}, c_{p})\}$ for any $c_{p}, c_{q}\in V, p \neq q$. Each arc $\overrightarrow{a}$ of $\overleftrightarrow{D}$ has a frequency, denoted by $f_{\overrightarrow{a}}$, which represents the fraction of digraphs in $S$ that contain the arc $\overrightarrow{a}$. Based on the arc frequencies, we employ the k-means algorithm [23] to partition all the arcs of $\overleftrightarrow{D}$ into three portions, denoted by $H$, $L$, and $M$, respectively of highest, lowest, and medium frequencies. Next, we incorporate a new digraph $D_{k_{-c}}$, where $k\in \{1,2,\dots ,n\} \backslash \{i,j\}$, and $c\in \{1,2\}$ into $S$ if

(1) $D_{k_{-c}}$ shares the most arcs with one of $S$, and to which subjects,
(2) $KL_{D_{k_{-c}}}(H,L)>KL_{0}$, where $KL_{D_{k_{-c}}}(H,L)$ is the Kullback–Leibler divergence score [24] between arcs $H$ and $L$ in $D_{k_{-c}}$,
  (3) \begin{align*}& KL_{D_{k_{-c}}}(H,L) = F_{H} \times \log\left(\frac{F_{H}}{F_{L}}\right) + F_{\overline{H}} \times \log\left(\frac{F_{\overline{H}}}{F_{\overline{L}}}\right),\end{align*}

where $F_{Z}=|Z\cap A(D_{k_{-c}})|/|Z|$, $F_{\overline{Z}}=|Z-A(D_{k_{-c}})|/|Z|$, with $Z\in \{H,L\}$. $KL_{0}$ is a threshold specified by users (default value: 0.3). According to the new $S$, we update $H$ by recalculating the arc frequencies $f_{\overrightarrow{a}}$. Repeat the process until $H$ remains unchanged for multiple consecutive iterations (see the theoretical basis for redefining $S$ by 1 and 2 in Supplementary Note).

**Step 3. Identifying a CMAT subdigraph.** In this step, we identify the CMAT subdigraph from the digraph $D=(V,A)$, where $V=\{c_{1},c_{2},\dots ,c_{m}\}$ and $A=H$. Conversely, the digraph $D$ can be interpreted as a noisy variant of the CMAT subdigraph, potentially incorporating redundant arcs or missing some arcs that should be part of the ideal CMAT structure, due to methodological limitations or data noise. Let $N^{+}_{D}(c)$ and $N^{-}_{D}(c)$ denote the out-neighborhood and in-neighborhood of vertex $c$ in digraph $D$. Specifically, $N^{+}_{D}(c) = \{ c_{i} \mid \forall c_{i} \in V, (c, c_{i}) \in A(D) \}$ and $N^{-}_{D}(c) = \{ c_{i} \mid \forall c_{i} \in V, (c_{i}, c) \in A(D) \}$. If the digraph $D$ follows an ideal CMAT structure, its partite sets can be sequentially ordered as $P_{1},P_{2},\dots ,P_{l}$, such that for any partite set $P_{i}$, each vertex $c$ in $P_{i}$ has $N^{+}_{D}(c)=\cup ^{i-1}_{j=1}P_{j}$ and $N^{-}_{D}(c)=\cup ^{l}_{j=i+1}P_{j}$, where $1<i<l$. Each vertex $c$ in $P_{1}$ only has $N^{-}_{D}(c)=\cup ^{l}_{j=2}P_{j}$, and each vertex $c$ in $P_{l}$ only has $N^{+}_{D}(c)=\cup ^{l-1}_{j=1}P_{j}$ (see an example in Fig. 1B). Leveraging this structural property, we iteratively identify all partite sets $P_{1},P_{2},\dots ,P_{l}$ from $D$. Since $D$ is noisy, we introduce two parameters, $\sigma $ and $\gamma $.

We initiate by selecting the vertex in $D$ with the highest in-degree and setting it as $c_{k}$. The vertex $c_{k}$ is used to locate the first partite set $P_{1}$, i.e. $ c_{k}\in P_{1}$. For each vertex $c$ in $D$ that has not yet been assigned to any of the identified partite sets, the correlation value $\gamma $ between $c$ and $c_{k}$ is calculated as follows: 


(4) \begin{align*}& \gamma(c,c_{k})=\frac{|N^{+}_{D}(c)\cap N^{+}_{D}(c_{k})|+|N^{-}_{D}(c)\cap N^{-}_{D}(c_{k})|}{|N_{D}(c_{k})|},\end{align*}

where $N_{D}(c)=N^{+}_{D}(c)\cap N^{-}_{D}(c)$. Vertices with $\gamma>\gamma _{0}$, where $\gamma _{0}\in [0,1]$ (default value: 0.85), are assigned to the initial partite set $P_{1}$. Next, we reset $c_{k}$ to locate the next partite set $P_{2}$. For each remaining unassigned vertex $c$, if it satisfies the condition $|N^{+}_{D}(c)\cap \cup ^{i-1}_{j=1}P_{j}|\geq \sigma * |\cup ^{i-1}_{j=1}P_{j}|$, where $\sigma \in [0,1]$ (default value: 0.75) and $i=2$ (corresponding to $P_{2}$), then $c$ is considered a candidate for the next $c_{k}$. Among all candidate vertices, the one with the highest in-degree is selected as the new $c_{k}$. Then repeat the above procedure to determine the remaining vertices in $P_{2}$. Following this way, we iteratively identify all partite sets $P_{1},P_{2},\dots ,P_{l}$ until no new $c_{k}$ can be used.

**Step 4. Determining the corresponding BTP-bicluster.** Given an error rate $e_{0}$, we extract all digraphs containing the CMAT subdigraph, allowing up to a fraction $e_{0}$ of missing arcs. We calculate the corresponding $P$-value $P$ (see Supplementary Note) and declare the CMAT subdigraph statistically significant if $P<P_{0}$, where $P_{0}$ is a predefined threshold. All digraphs containing the CMAT subdigraph correspond to genes exhibiting a BTP-pattern in the partition $ P_{1},P_{2},\dots ,P_{l}$. Specifically, for each gene $g_{i}$, if its digraph $D_{i_{-1}}$ contains the CMAT subdigraph, it has the BTP-pattern $min\{a_{i,j}:c_{j}\in P_{d} \}>max\{a_{i,j}:c_{j}\in P_{d+1} \},d=1,2,\dots ,l-1$. Conversely, if $D_{i_{-2}}$ contains the CMAT subdigraph, it has $max\{a_{i,j}:c_{j}\in P_{d} \}<min\{a_{i,j}:c_{j}\in P_{d+1} \},d=1,2,\dots ,l-1$.

**Step 5. Iteratively outputting all BTP-biclusters.** We reset $S$ to include a new pair of digraphs for identifying another CMAT subdigraph. The pair must meet two criteria: (i) at least one of its digraphs does not contain any previously identified CMAT subdigraphs, and (ii) the pair has not been used together to search for the same CMAT subdigraphs. Repeat **Steps 2–4** until no new pair can be used to initialize $S$.

## Results

### Datasets

In this research, we evaluated the performance of the compared tools using both simulated and real datasets. For the simulated data, each row of the background matrices was generated from a Gaussian distribution *N*(1,1), following previous protocols [7, 10]. In addition, we also employed an alternative method for generating background matrices, mimicking real gene expression data. The background matrix contains 10 000 genes and 50 conditions, and each row was sampled from one of five distributions—Normal, Gamma, Bimodal, Cauchy, and Lognormal—based on the findings of De Torrenté *et al*. [25], followed by z-score normalization. To preserve the original distribution of the background matrices, we applied the bicluster implantation approach by Wang *et al*. [6]. For shifting, scaling, and combined shifting-scaling biclusters, the shifting parameters were drawn from the set {−1, −0.8, −0.5, −0.2, 0, 0.2, 0.5, 0.8, 1}, and the scaling parameters from {−3, −2, −1, 1, 2, 3}. Overlapping biclusters were constructed such that genes adhered to the BTP-patterns across all biclusters to which they belonged. The noise was introduced into BTP-biclusters by replacing certain expression values in each row with new samples from the same distribution, causing genes to deviate from the intended BTP-pattern. Considering the expression trend of a gene across two conditions as the basic unit, the noise level for each gene was determined by the fraction of basic units in which the gene’s expression deviated from the expected pattern. The highest noise level among all genes within a bicluster was defined as the noise level of the BTP-bicluster. Following the approach of Orzechowski *et al*. [9], we introduced normally distributed data fluctuations *N*(0, $\sigma $) with $\sigma $ values of 0.1, 0.2, and 0.3. For the real datasets, we used six gene expression datasets from four diseases: type 2 diabetes (T2D) [26], colorectal cancer (CC) [27], hepatocellular carcinoma (HC) [28], and breast cancer (BC) [29], obtained from Gene Expression Omnibus (accession numbers: GSE77943, GSE178145, GSE6764, and GSE46141). The T2D dataset includes expression profiles from three distinct tissues: adipose, liver, and pancreas. We downloaded the processed datasets and selected the 20 000 genes with the highest standard deviations as input. A pseudo count of 1 was added to the expression counts, followed by log2 transformation and z-score normalization for each gene.

### Evaluation criterion

When applied to the simulated dataset, we adopted two widely used metrics, Recovery and Relevance [30], following previous studies [10]. The two metrics provide complementary assessments of the agreement between detected and genuine biclusters, denoted by $D$ and $G$, 


(5) \begin{align*} & Recovery=\frac{1}{|G|}\sum\limits_{b_{1}\in G}\max\limits_{b_{2}\in D}\frac{|b_{1}\cap b_{2}|}{|b_{1}\cup b_{2}|} \end{align*}

(6) \begin{align*} & Relevance=\frac{1}{|D|}\sum\limits_{b_{2}\in D}\max\limits_{b_{1}\in G}\frac{|b_{1}\cap b_{2}|}{|b_{1}\cup b_{2}|} \end{align*}

To facilitate a simultaneous comparison of algorithms on both Recovery and Relevance, we used the Performance metric from DESMOND, defined as the geometric mean of the Relevance and Recovery scores. In the real datasets, there is no golden benchmark to validate the accuracy of biclustering algorithms. Haynes *et al*. [31] recommended using data-driven gene sets. For example, Rose *et al*. [20] selected the KEGG pathways including the word ”cancer,” ”carcinoma,” or ”tumor” for biclustering algorithm comparison on the cancer datasets. Following this way, we selected the KEGG pathways related to specific diseases as benchmarks (see Table S1) and compared algorithms based on the number of enriched pathways. To mitigate biases in the comparative analysis of various tools, we explored diverse parameter configurations to optimize each tool’s performance across datasets. Supplementary Table 1 details the parameter settings for both synthetic and real datasets, with values chosen proximate to default configurations. For TranBic, we tested four main threshold parameters in the real datasets (see Supplementary Note for their impact on the results).

### Test of the algorithm on artificial datasets

**Figure 3:**
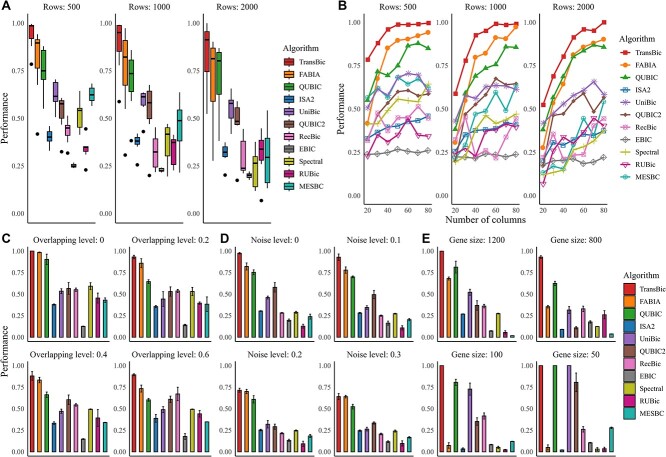
Comparison of the algorithms in identifying BTP-biclusters.

*TransBic outperforms the existing methods in identifying the BTP-biclusters.* We comprehensively assess the efficacy of TransBic in identifying BTP-biclusters, considering five distinct influencing factors: (i) the number of row features in the background matrices, (ii) the number of column features, (iii) the degree of overlap among biclusters, (iv) noise levels, and (v) the gene sizes of BTP-biclusters. The background matrices for the first four scenarios were generated using a Gaussian distribution *N*(1,1), while the fifth scenario was designed to mimic real gene expression datasets, with gene sizes determined based on the actual sizes of GO terms and KEGG pathways, ranging from tens to thousands. As illustrated in Fig. 3, TransBic consistently demonstrated superior performance across varying factors (see details in the Supplementary Note).

**Figure 4:**
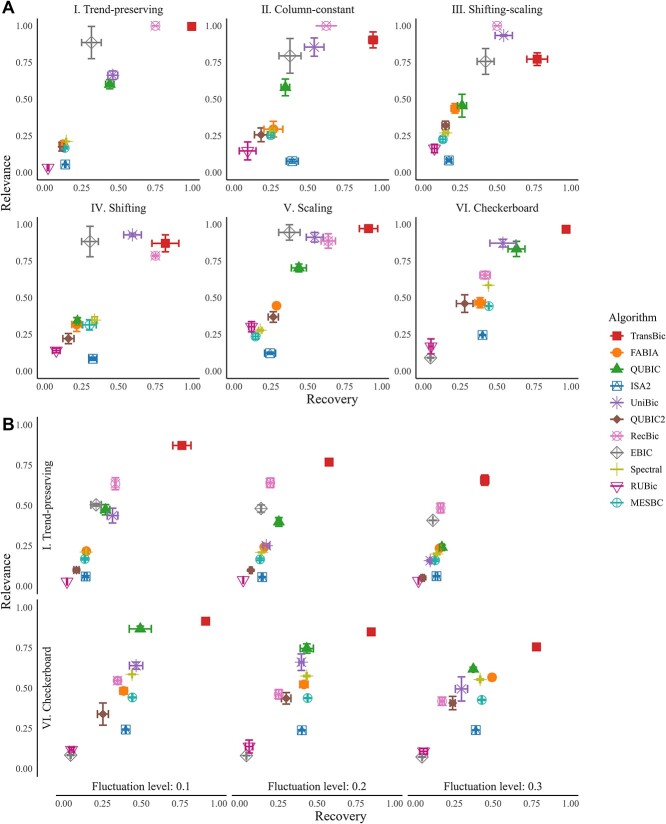
A. Comparisons of the algorithms in identifying different patterns. B. Comparisons of the algorithms on datasets in identifying patterns against different fluctuation levels: 0.1, 0.2, and 0.3.

*TransBic outperforms the existing methods in identifying different patterns, especially against data fluctuations.* In this section, we compared TransBic and other algorithms in identifying biclusters with the following patterns: column-constant, shifting-scaling, shifting, scaling, checkerboard, order-preserving, and trend-preserving. The order-preserving pattern is not tested here, as its underlying pattern is trend-preserving. As illustrated in Fig. 4A, TransBic achieved the best overall performance compared with other algorithms. Only in terms of relevance, TransBic demonstrated comparable or slightly lower performance relative to RecBic and UniBic. When data fluctuations are introduced (Fig. 4B), TransBic performs better than other algorithms across all fluctuation levels (see details in the Supplementary Note). Given the above patterns could be classified as trend-preserving and checkerboard, we only assess algorithmic performance for the two patterns across different fluctuation levels.

### Test of the algorithm on real datasets

*TransBic outperforms the existing methods in identifying disease-related pathways.* On real datasets, we verified that TransBic can effectively identify disease-related pathways. Specifically, we conducted KEGG enrichment analysis on the outputs of different algorithms (see Supplementary Note for details), and recorded the number of enriched pathways for each dataset and across all datasets (see Table 1). The results show that TransBic identified the most disease-associated pathways (see Supplementary Fig. S10 for details). Among the top four algorithms—TransBic, UniBic, QUBIC2, and QUBIC—TransBic does not dominate in the number and size of biclusters (see Tables S2 and S3 for details). This indicates that the superiority of TransBic is not due to the identification of more or larger biclusters. We further integrated the results of UniBic, QUBIC, and QUBIC2 using MoSBi and found that the performance of the integrated results remains inferior to that of TransBic. In conclusion, TransBic demonstrates effectiveness in identifying FGMs of biological relevance.

**Table 1:** Enrichment results of the algorithms on the KEGG pathways related to corresponding diseases. The entry refers to the number of enriched pathways/the number of all disease-related pathways.

${}_{\mathrm{\bf Algorithm}}\Big\backslash{}^{\mathrm{\bf Dataset}}$	T2D (adipose)	T2D (liver)	T2D (pancreas)	CC	HC	BC	ALL
TransBic	**12/13**	**12/13**	**7/8**	**10/10**	**12/12**	**9/9**	**62/65 (0.95)**
FABIA	7/13	8/13	6/8	8/10	11/12	8/9	48/65 (0.74)
QUBIC	11/13	7/13	5/8	**10/10**	**12/12**	**9/9**	54/65 (0.83)
ISA2	10/13	9/13	2/8	9/10	**12/12**	**9/9**	51/65 (0.78)
UniBic	**12/13**	10/13	**7/8**	**10/10**	**12/12**	**9/9**	60/65 (0.92)
QUBIC2	11/13	**12/13**	6/8	7/10	**12/12**	**9/9**	57/65 (0.88)
RecBic	10/13	9/13	5/8	6/10	**12/12**	**9/9**	51/65 (0.78)
EBIC	8/13	7/13	6/8	0/10	11/12	4/9	36/65 (0.55)
Spectral	9/13	5/13	4/8	9/10	9/12	7/9	43/65 (0.66)
BiCoN	4/13	1/13	1/8	0/10	4/12	3/9	13/65 (0.2)
DESMOND	9/13	7/13	2/8	**10/10**	**12/12**	**9/9**	49/65 (0.75)
RUBic	1/13	0/13	0/8	0/10	9/12	7/9	17/65 (0.26)
MESBC	7/13	3/13	3/8	8/10	9/12	7/9	37/65 (0.57)
MoSBi	4/13	5/13	2/8	9/10	9/12	7/9	36/65 (0.55)

The highest score in each column is highlighted in bold.

**Figure 5:**
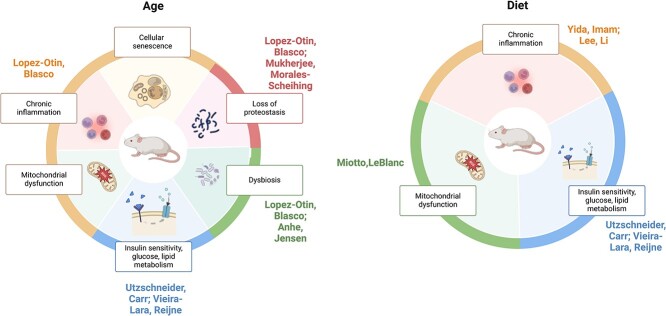
The biological processes identified by TransBic that are influenced by age and diet.

*TransBic identifies biological processes affected by disease risk factors.* Using the T2D expression datasets, we validated that TransBic can identify biological processes affected by certain risk factors. The dataset comprises gene expression profiles from three tissues—adipose, liver, and pancreas—collected from outbred mice. It is designed to investigate the impact of two critical risk factors, age (weeks 1, 9, and 18) and diet (regular chow and high-fat diet), on the development of type 2 diabetes. For the identified BTP-biclusters, we calculated Spearman’s rank correlation coefficients [32] between their specific expression trends and the development of age and diet (see Supplementary Note for details). For those biclusters with Spearman correlation scores above 0.5, we performed the GOBP enrichment analysis. As shown in Fig. 5, TransBic identifies significant biological processes affected by age or diet, coincident with those from previous literature [33–39] (see details in Table S4).

*Ablation study.* To simultaneously identify FGMs and their specific up- and down-regulation patterns, TransBic transforms the problem into the detection of CMAT subgraphs. In this process, TransBic consists of two key components: Step 2 and Step 3. In Step2, TransBic designs a heuristic search method to outline the approximate range of CMAT subgraphs, represented as high-frequency digraphs. And in Step 3, TransBic further refines and adjusts these high-frequency digraphs to match the structural characteristics of CMAT subgraphs. To evaluate the effectiveness of the design of our TransBic, we conducted an experiment that compared it with two ablation settings, including (i) replacing Step 2 with the greedy search used in the previous trend-preserving algorithms, and (ii) removing Step 3 entirely. Applying them, respectively, or simultaneously on the real datasets gives us four different combinations. As shown in Table 2, Step 2 contributes the most to the performance, and Step 3 further increases the metric score, collectively resulting in a substantial enhancement. This indicates that both Step 2 and Step 3 are crucial in developing an optimized biclustering model, leading to precise identification of meaningful local patterns.

**Table 2:** Ablation study on key components: Steps 2 and 3. Ablation setting “(i)” is designed for Step 2, and “(ii)” is for Step 3. “a”, “b”, “c”, and “d” denote four different combinations.

${}_{\mathrm{\bf Combination}}\Big\backslash{}^{\mathrm{\bf Ablation\ setting}}$	(i)	(ii)	T2D (adipose)	T2D (liver)	T2D (pancreas)	CC	HC	BC	ALL
a	$\checkmark $	$\checkmark $	3/13	1/13	2/8	5/10	0/12	0/9	11/65 (0.17)
b	$\times $	$\checkmark $	10/13	6/13	**7/8**	9/10	11/12	7/9	50/65 (0.77)
c	$\checkmark $	$\times $	9/13	8/13	**7/8**	**10/10**	7/12	2/9	43/65 (0.66)
d	$\times $	$\times $	**12/13**	**12/13**	**7/8**	**10/10**	**12/12**	**9/9**	**62/65 (0.95)**

The highest score in each column is highlighted in bold.

## Discussion

In this work, we introduced a novel BTP-pattern, which generalizes all existing biologically significant local patterns, except for the row-constant pattern. We also developed TransBic, a method specifically designed to identify biclusters that exhibit BTP-patterns (referred to as BTP-biclusters). By detecting these BTP-biclusters, we successfully identified biclusters exhibiting distinct local patterns. In tests on synthetic datasets, TransBic outperformed 10 state-of-the-art biclustering algorithms in identifying distinct patterns and showed greater robustness to data fluctuations. Experiments on real gene expression datasets further showed that the BTP-biclustering model enhances the ability of algorithms to identify biologically significant FGMs. For example, TransBic identified the most disease-related KEGG pathways. Furthermore, TransBic enables the application of trend-preserving biclustering to identify biological processes affected by disease risk factors. Overall, TransBic is a powerful tool for analyzing gene expression datasets, but it has some limitations: its high computational complexity makes it unsuitable for analyzing datasets with large numbers of conditions (see Supplementary Note). Future research will focus on developing more efficient biclustering algorithms and applying them to larger and noisier datasets, such as single-cell and spatial transcriptomics data.

**Key Points**

- Introduce a novel pattern, BTP-pattern, generalizing distinct meaningful patterns.
- Develop a new algorithm, TransBic, to identify distinct patterns.
- Extend the application of trend-preserving biclustering in analyzing expression datasets.

## Supplementary Material

supplementary_TransBic_bbaf050

Supplementary_Table_1_bbaf050

## Data Availability

Code: https://github.com/LiJing-source-coder/TransBic. Data: https://zenodo.org/uploads/13777956.
