# Supplementary material for: TransBic: bucket trend-preserving biclustering for finding local and interpretable expression patterns
Source: Brief Bioinform. 2025 Feb 5;26(1):bbaf050. doi: 10.1093/bib/bbaf050 (PMC11794469; doi:10.1093/bib/bbaf050)
Supplement: supplementary_TransBic_bbaf050 [file supplementary_transbic_bbaf050.docx]

**Supplementary Information**

Jing Li, Qinglin Mei, Chaoxia Yang, Naibo Zhu, and Guojun Li

Supplementary Tables:

Table S1. KEGG pathways associated with Type 2 Diabetes (T2D), Colorectal Cancer (CC), Hepatocellular Carcinoma (HC), and Breast Cancer (BC). For T2D, three different tissues are considered: adipose and liver, which are associated with insulin resistance, and the pancreas, which is associated with insulin secretion.

| Datasets | Pathways | Entry | | Website |
| --- | --- | --- | --- | --- |
| T2D(adipose) | Type 2 diabetes mellitus and related pathways;  Insulin resistance and related pathways | mmu04930; mmu04210;  mmu04910;  mmu04920;  mmu04950;  mmu04931  mmu00010; | mmu00061;  mmu00514;  mmu00520;  mmu04150;  mmu04151;  mmu04668; | <https://www.kegg.jp/entry/mmu04930>;  <https://www.kegg.jp/entry/mmu04931>; |
| T2D(liver) | Type 2 diabetes mellitus and related pathways;  Insulin resistance and related pathways | mmu 04930; mmu04210;  mmu04910;  mmu04920;  mmu04950;  mmu04931  mmu00010; | mmu00061;  mmu00514;  mmu00520;  mmu04150;  mmu04151;  mmu04668; | <https://www.kegg.jp/entry/mmu04930>;  <https://www.kegg.jp/entry/mmu04931>; |
| T2D(pancreas) | Type 2 diabetes mellitus and related pathways;  Insulin secretion and related pathways | mmu 04930;  mmu04210;  mmu04910;  mmu04920; | mmu04950;  mmu04911;  mmu00020;  mmu04020; | <https://www.kegg.jp/entry/mmu04930>;  <https://www.kegg.jp/entry/mmu04911>; |
| CC | Colorectal cancer and related pathways | mmu05210;  mmu04010;  mmu04012;  mmu04110;  mmu04115; | mmu04150;  mmu04151;  mmu04210;  mmu04310;  mmu04350; | <https://www.kegg.jp/entry/pathway+mmu05210>; |
| HC | Hepatocellular carcinoma and related pathways | hsa05225;  hsa04010;  hsa04020;  hsa04110;  hsa04115;  hsa04151; | hsa04310;  hsa04350;  hsa04932;  hsa04936;  hsa05160;  hsa05161; | <https://www.kegg.jp/entry/hsa05225>; |
| BC | Breast cancer and related pathways | hsa05224;  hsa03440;  hsa04010;  hsa04110;  hsa04115; | hsa04151;  hsa04310;  hsa04330;  hsa04915; | <https://www.kegg.jp/entry/hsa05224>; |

Table S2. The number of biclusters identified by all the compared algorithms across different datasets: T2D (adipose), T2D (liver), T2D (pancreas), CC, HC, and BC.

| **Datasets**  **Algorithm** | **T2D**  **(adipose)** | **T2D**  **(liver)** | **T2D**  **(pancreas)** | **CC** | **HC** | **BC** |
| --- | --- | --- | --- | --- | --- | --- |
| **TransBic** | 48 | 42 | 52 | 34 | 180 | 46 |
| **FABIA** | 16 | 16 | 18 | 10 | 26 | 36 |
| **QUBIC** | 97 | 41 | 33 | 48 | 100 | 92 |
| **ISA2** | 43 | 41 | 3 | 9 | 129 | 201 |
| **UniBic** | 65 | 96 | 74 | 100 | 100 | 100 |
| **QUBIC2** | 83 | 99 | 98 | 28 | 95 | 100 |
| **RecBic** | 6 | 17 | 14 | 6 | 100 | 100 |
| **EBIC** | 100 | 100 | 29 | 1 | 100 | 100 |
| **Spectral** | 4 | 4 | 4 | 4 | 3 | 4 |
| **BiCoN** | 2 | 2 | 1 | 2 | 2 | 2 |
| **DESMOND**  **RUBic**  **MESBC**  **MoSBi** | 22  18  3  11 | 30  0  2  14 | 31  0  2  12 | 52  0  2  10 | 206  83  7  10 | 48  90  9  20 |

Table S3. The gene sizes of biclusters identified by all the compared algorithms across different datasets: T2D (adipose), T2D (liver), T2D (pancreas), CC, HC, and BC. Values are presented as the minimum gene size - maximum gene size (average size).

| **Datasets**  **Algorithm** | **T2D**  **(adipose)** | **T2D**  **(liver)** | **T2D**  **(pancreas)** | | **CC** | **HC** | **BC** |
| --- | --- | --- | --- | --- | --- | --- | --- |
| **TransBic** | 172-4719  (1522.50) | 177-5630  (1662.76) | 130-4571  (1418) | 234-10318  (2106.09) | | 31-2866  (546.04) | 63-1655  (643.57) |
| **FABIA** | 5300-9509  (7677.44) | 5528-10468  (7466.38) | 922-5907  (2225.78) | 8695-12913  (11357.60) | | 2702-9374  (5917.00) | 2437-9012  (4560.64) |
| **QUBIC** | 718-3902  (1702.44) | 257-1451  (627) | 526-2257  (1142.27) | 21-12594  (2604.90) | | 623-4381  (1774.52) | 28-886  (227.13) |
| **ISA2** | 98-7182  (2400.19) | 20-6990  (1477.88) | 1634-6607  (3311.33) | 1104-7220  (3627.67) | | 62-6677  (1657.14) | 70-6264  (1503.60) |
| **UniBic** | 90-4508  (2236.23) | 80-4754  (1410.18) | 36-11495  (2446.18) | 363-9250  (2133.88) | | 66-4225  (637.32) | 5-4406  (308.48) |
| **QUBIC2** | 5-359  (41.25) | 5-225  (23.99) | 6-759  (53.44) | 8-1798  (137) | | 22-1585  (263.81) | 12-1249  (106.95) |
| **RecBic** | 24-188  (83.83) | 58-352  (172.71) | 6019-11107  (9073.07) | 10740-12103  (11423.33) | | 5660-8445  (6310.29) | 136-1716  (515.6) |
| **EBIC** | 304-2135  (972.88) | 390-2396  (518.84) | 464-4496  (3048.90) | 737  (737) | | 520-4113  (1884.77) | 253-2572  (792.26) |
| **Spectral** | 3919-6848  (5000.00) | 4889-5082  (5000.00) | 2887-6923  (5000.00) | 4477-5939  (5000.00) | | 5245-9260  (6666.67) | 4097-5558  (5000.00) |
| **BiCoN** | 9-22  (15.50) | 5-6  (5.50) | 14  (14.00) | 12  (12.00) | | 5  (5.00) | 5  (5.00) |
| **DESMOND**  **RUBic**  **MESBC**  **MoSBi** | 5-235  (32.27)  5-66  (14.89)  3846-4364  (4112)  9-4448  (1518.55) | 5-217  (21.97)  0  8545-11455  (10000)  16-9386  (2574.71) | 5-71  (15.87)  0  4888-8187  (6537.5)  21-6835  (1055.33) | 5-366  (34.37)  0  7250-7576  (7413)  47-16135  (3390) | | 5-429  (31.8)  10-344  (87.14)  832-1457  (1105.43)  923-14469  (5897.40) | 5-243  (23.52)  5-1082  (87.17)  699-2647  (1870.566)  23-8571  (1046.75) |

**Table S4.** The biological processes identified by TransBic that are influenced by age and diet. Representative Gene Ontology Biological Processes (GOBPs) are listed to support the conclusions. The symbol "/" indicates that the association between the biological process in that row and the risk factor in that column is not supported by the available literature.

| Biological process | Tissue | Representative GOBP (age>0.5) | Representative GOBP (diet>0.5) | Evidence-based literature |
| --- | --- | --- | --- | --- |
| Insulin sensitivity and glucose, lipid metabolism | Adipose | Response to insulin  Glucose metabolic process  Glucose homeostasis  Lipid catabolic process  Lipid homeostasis  Fatty acid metabolic process | Lipid biosynthetic process  Regulation of lipid metabolic process  Fatty acid metabolic process | Utzschneider, Carr [1]  Vieira-Lara, Reijne [2] |
|  | Liver | Response to insulin  Glucose catabolic process  Intracellular glucose homeostasis  Lipid homeostasis  Cellular response to lipid | Cellular response to lipid |  |
|  | Pancreas | Response to insulin  Intracellular glucose homeostasis  Cellular lipid catabolic process  Fatty acid metabolic process | Regulation of insulin secretion involved in cellular response to glucose stimulus  Insulin metabolic process  Glucose catabolic process |  |
| Chronic inflammation | Adipose | Regulation of inflammatory response  Immune effector process  Response to tumor necrosis factor  Positive regulation of interleukin-1 beta production  Interleukin-6 production  Macrophage activation involved in immune response | Regulation of acute inflammatory response  Activation of immune response  Response to interleukin-1 | López-Otín, Blasco [3]  Yida, Imam [4]  Lee, Li [5] |
|  | Liver | Acute inflammatory response to antigenic stimulus  Activation of immune response  Tumor necrosis factor production  Response to interleukin-1  Interleukin-1-mediated signaling pathway | Acute inflammatory response to antigenic stimulus  Activation of immune response  Response to interleukin-1  Interleukin-1-mediated signaling pathway  Tumor necrosis factor production |  |
|  | Pancreas | Regulation of inflammatory response  Activation of immune response;  Tumor necrosis factor production  Interleukin-1 beta production  Interleukin-6 production | Positive regulation of inflammatory response to antigenic stimulus |  |
| Mitochondrial dysfunction | Adipose | Regulation of mitochondrial membrane permeability involved in apoptotic process  Mitochondrial outer membrane permeabilization involved in programmed cell death  Apoptotic mitochondrial changes  Response to reactive oxygen species  Response to hypoxia  Intrinsic apoptotic signaling pathway in response to oxidative stress  Oxidative phosphorylation  Positive regulation of fatty acid oxidation  Negative regulation of ATP-dependent activity  ATP biosynthetic process | Mitochondrial gene expression  Mitochondrial translation  Intrinsic apoptotic signaling pathway in response to oxidative stress  Aerobic respiration  Tricarboxylic acid cycle  Mitochondrial ATP synthesis coupled electron | López-Otín, Blasco [3]  Miotto, LeBlanc [6] |
|  | Liver | Regulation of ATP metabolic process  Regulation of mitochondrial fusion  Mitochondrial gene expression  Response to hypoxia | Regulation of ATP metabolic process  Response to hypoxia |  |
|  | Pancreas | Positive regulation of mitochondrial fission  Positive regulation of ATP biosynthetic process  Response to hypoxia  Positive regulation of ATP-dependent activity  Fatty acid oxidation  Response to oxidative stress  Intrinsic apoptotic signaling pathway in response to oxidative stress  Cellular response to reactive oxygen species  Regulation of execution phase of apoptosis |  |  |
| Loss of proteostasis | Adipose | Protein stabilization  Response to amyloid-beta | / | Mukherjee, Morales-Scheihing [7]  López-Otín, Blasco [3] |
|  | Liver | Protein folding  Response to amyloid-beta  Response to misfolded protein  Regulation of protein stability  Protein stabilization | / |  |
|  | Pancreas | Cellular response to amyloid-beta  Protein folding  Response to topologically incorrect protein  Amyloid-beta formation  Response to misfolded protein  Protein stabilization  Amyloid precursor protein catabolic process | / |  |
| Cellular senescence | Pancreas | Cellular senescence | / | López-Otín, Blasco [3] |
| Dysbiosis (gut microbiome) | Pancreas | Defense response to Gram-positive bacterium | / | López-Otín, Blasco [3]  Anhê, Jensen [8] |

**Table S5.** Running time of the tools on synthetic datasets with different row numbers. Specifically, we generated datasets used for testing with 2000, 4000, 6000, 8000, 10000 rows and 50 columns. Their background matrices were generated following a normal distribution *N*(1, 1). For every dataset, we implanted five BTP-biclusters with their respective sizes: (200, 200) × (1/10, 1/10, 1/10), (200, 150) × (1/5, 1/5), (200, 200) × (1/5, 2/5), (300, 0) × (1/6, 1/6, 2/6) and (200, 0) × (1/5, 1/5, 1/5, 1/5). All tools were run on a server (with a CPU Intel(R) Core (TM) i7-9750H, 2.60GHz, and RAM 24GB) with their default parameters. The number of output biclusters was set to 5 for these tools if needed. For RUBic, it consistently outputs tens or even hundreds of thousands of biclusters, making it unsuitable for comparison. Other algorithms, limited by the real dataset, are not included for comparison. As shown in Table S5, TransBic spends more running time compared with other algorithms on the datasets with 6000, 8000, and 10000 rows, but acceptable.

| Row number  **Algorithm** | 2000 | 4000 | 6000 | 8000 | 10000 |
| --- | --- | --- | --- | --- | --- |
| TransBic | 0m47.882s | 2m32.796s | **5m38.537s** | **11m9.683s** | **18m45.568s** |
| FABIA | 0m1.202s | 0m2.369s | 0m3.591s | 0m4.941s | 0m6.193s |
| QUBIC | 0m0.451s | 0m1.122s | 0m2.369s | 0m4.216s | 0m6.944s |
| ISA2 | 0m1.697s | 0m2.483s | 0m4.366s | 0m6.347s | 0m7.637s |
| UniBic | 0m1.587s | 0m5.301s | 0m12.721s | 0m24.621s | 0m38.451s |
| QUBIC2 | 0m57.295s | 1m55.027s | 3m0.972s | 3m52.721s | 4m48.457s |
| RecBic | 0m21.532s | 0m33.066s | 0m44.509s | 0m53.360s | 1m9.963s |
| EBIC | **4m51.510s** | **4m29.707s** | 5m17.208s | 5m47.689s | 6m33.726s |
| Spectral | 0m0.943s | 0m2.417s | 0m4.619s | 0m7.120s | 0m9.619s |
| MESBC | 0m0.941s | 0m2.276s | 0m4.341s | 0m7.251s | 0m11.102s |

Table S6. Running time of the tools on real datasets. All tools were run on a server (with a CPU Intel(R) Core (TM) i7-10875H, 2.30GHz, and RAM 32GB) with their default parameters. The number of output biclusters was set to 100 for these tools if needed. As shown in Table S6, TransBic spends more running time compared with other algorithms on the HC, and BC datasets, but acceptable.

| Dataset  **Algorithm** | T2D  (adipose) | T2D  (liver) | T2D  (pancreas) | CC | HC | BC |
| --- | --- | --- | --- | --- | --- | --- |
| TransBic | 15m57.250s | 12m59.438s | 15m10.772s | 10m38.864s | **2h23m33.245s** | **4h22m4.872s** |
| FABIA | 17.562s | 17.780s | 17.152s | 15.182s | 38.398s | 45.052s |
| QUBIC | 40.983s | **1h19m6.640s** | **49m15.533s** | 55.848s | 2m31.525s | 2m14.186s |
| ISA2 | **21m6.982s** | 25m19.528s | 21m50.721s | **17m19.871s** | 41m32.594s | 48m7.078s |
| UniBic | 1m53.499s | 2m1.585s | 2m28.548s | 3m0.122s | 1m49.59s | 1m7.080s |
| QUBIC2 | 15.596s | 15.664s | 8.122s | 15.499s | 1m6.903s | 45.163s |
| RecBic | 11.722s | 12.883s | 13.529s | 7.222s | 7m58.249s | 15m58.191s |
| EBIC | 6m41.618s | 5m24.681s | 3m57.051s | 3m33.193s | 5m18.450s | 4m32.067s |
| Spectral | 8.229s | 8.222s | 8.628s | 7.738s | 8.900s | 9.049s |
| MESBC | 31.279s | 34.439s | 32.521s | 24.968s | 1m0.380s | 1m7.908s |
| BiCoN | 26.906s | 23.810s | 48.378s | 52.612s | 54.152s | 1m22.297s |
| DESMOND | 38.35s | 6.58s | 9s | 6.2s | 12m4.180s | 4m41.91s |
| MoSBi | 1m10.786s | 21.109s | 44.213s | 1m11.479s | 55.515s | 8.013s |

Supplementary Figures:


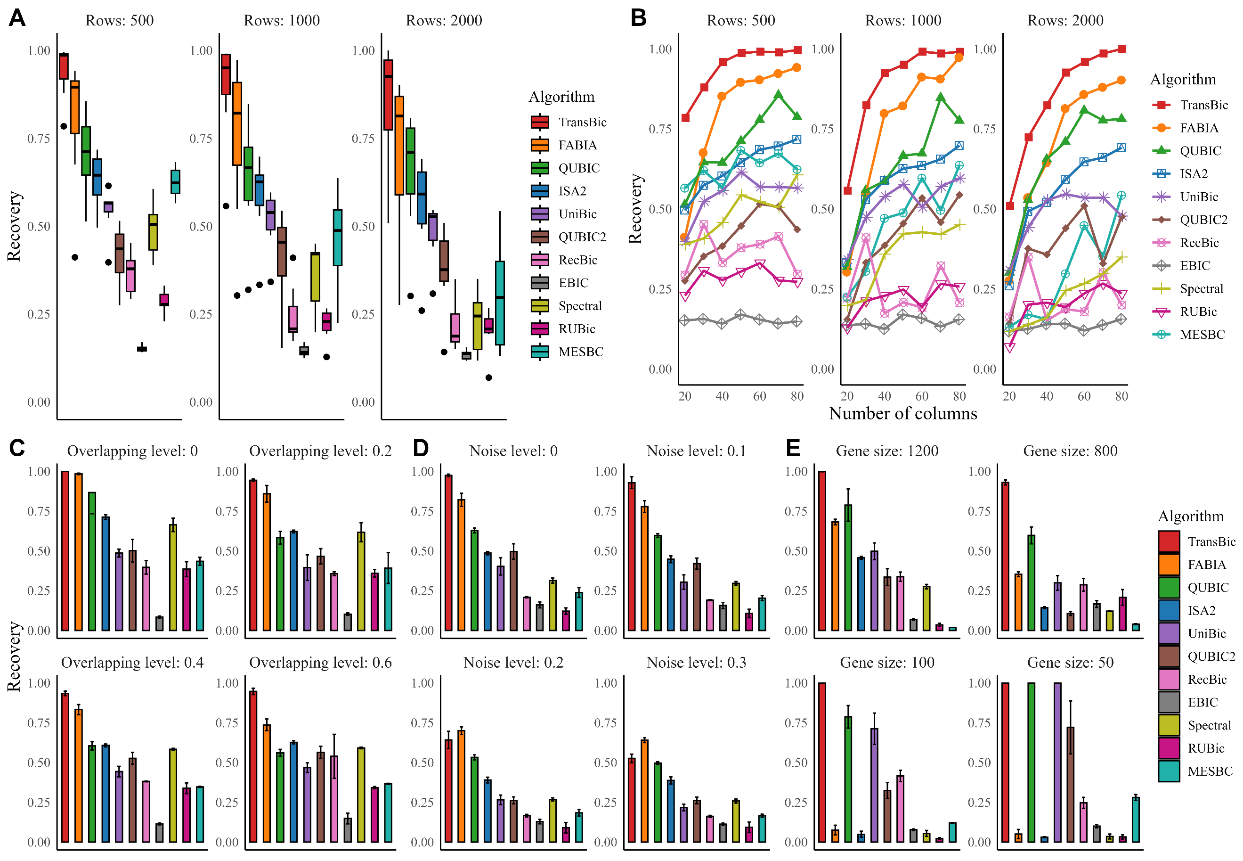


**Fig. S2.** Comparison of the tools in identifying BTP-biclusters in terms of Recovery scores. A. Comparisons of the tools in the background expression matrices with three different number of rows: 500, 1000 and 2000. B. Comparisons of the tools in the background expression matrices with different number of rows: 500, 1000, and 2000, and different number of columns: 20, 30, 40, 50, 60, 70, and 80. C. Comparisons of the tools on datasets with different overlapping levels: 0, 0.2, 0.4 and 0.6. D. Comparisons of the tools on datasets with different noise levels: 0, 0.1, 0.2 and 0.3. E. Comparisons of the tools on synthetic datasets mimicking real gene expression data. Implanted biclusters have four different sizes of genes: 1200, 800, 100, and 50.


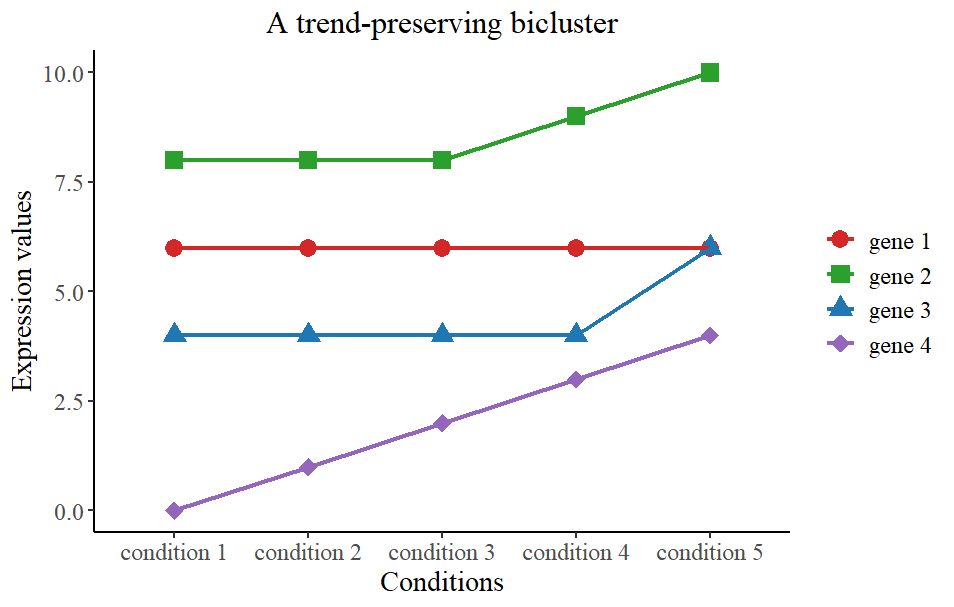


**Fig. S1.** An example of trend-preserving biclusters identified by previous algorithms, consisting of genes 1, 2, 3, 4 and conditions 1, 2, 3, 4, 5. Gene 1 maintains a constant expression level, and gene 2 gradually upregulates from condition 3 to condition 4, 5. Gene 3 upregulates from condition 4 to 5, and gene 4 steadily upregulates from condition 1 to 5. Although the genes do not upregulate simultaneously, previous algorithms still group them into the same bicluster.

**
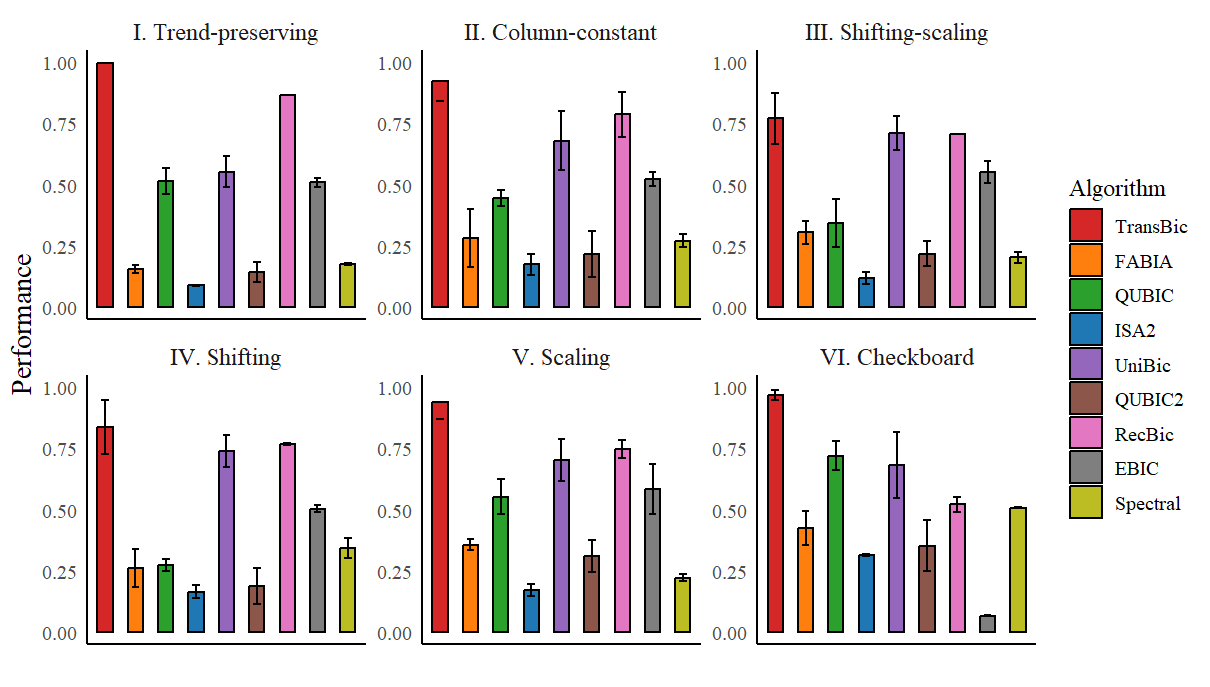
**

**Fig. S4.** Comparisons of the tools on the six datasets containing various bicluster patterns: trend-preserving, column-constant, shift-scale, shift, scale and constant-upregulated patterns in terms of Performance scores.


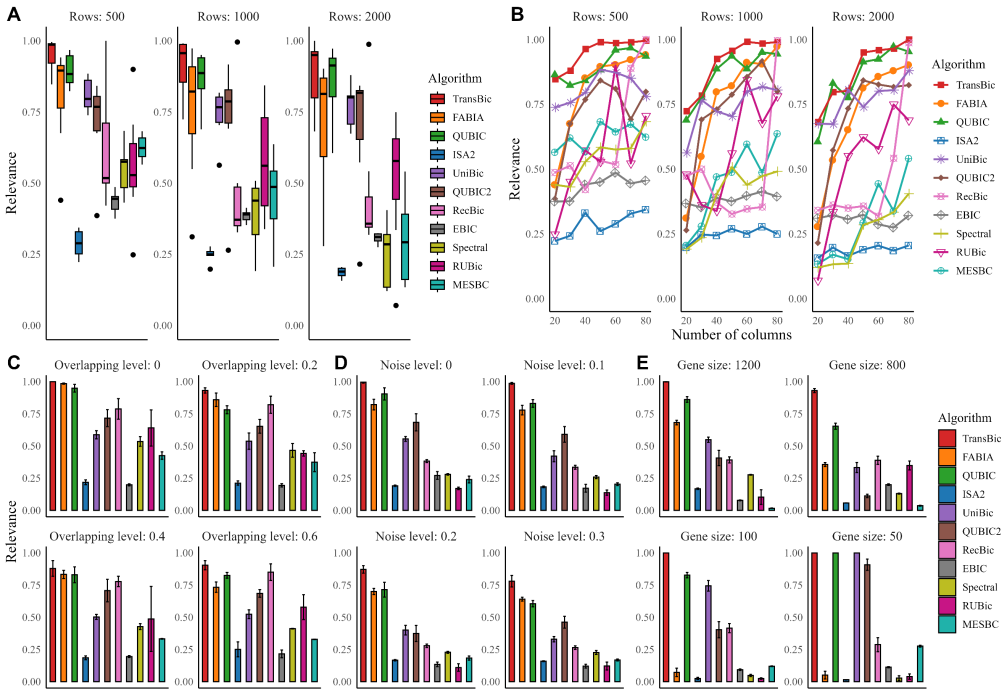


**Fig. S3.** Comparison of the tools in identifying BTP-biclusters in terms of Relevance scores. A. Comparisons of the tools in the background expression matrices with three different number of rows: 500, 1000 and 2000. B. Comparisons of the tools in the background expression matrices with different number of rows: 500, 1000, and 2000, and different number of columns: 20, 30, 40, 50, 60, 70, and 80. C. Comparisons of the tools on datasets with different overlapping levels: 0, 0.2, 0.4 and 0.6. D. Comparisons of the tools on datasets with different noise levels: 0, 0.1, 0.2 and 0.3. E. Comparisons of the tools on synthetic datasets mimicking real gene expression data. Implanted biclusters have four different sizes of genes: 1200, 800, 100, and 50.

**
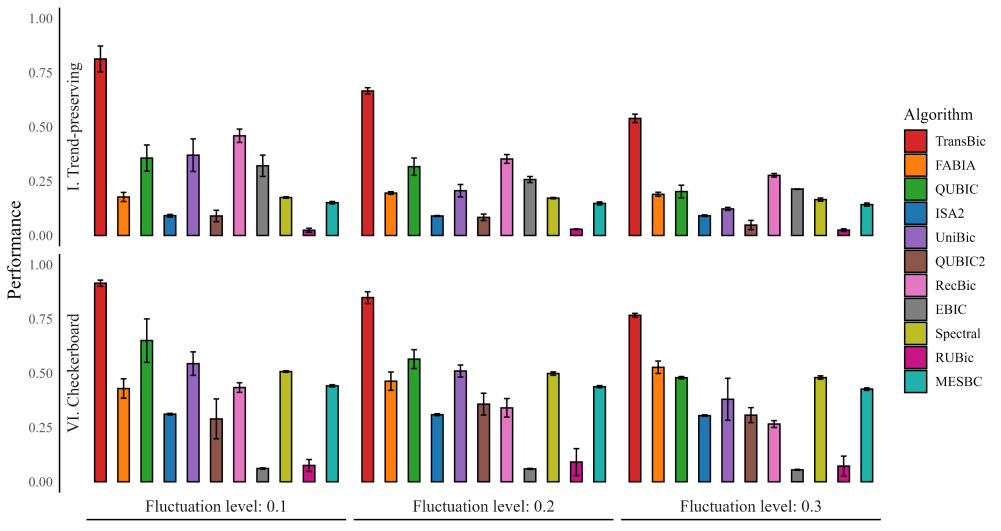
**

**Fig. S7.** Comparisons of the tools on datasets with different fluctuation levels: 0.1, 0.2 and 0.3 for trend-preserving and constant-upregulated biclusters respectively in terms of Performance scores.

**
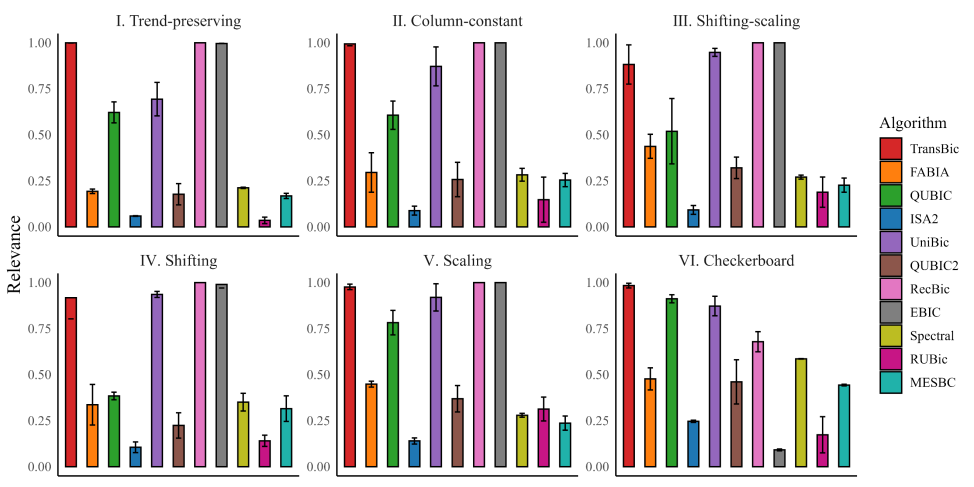
**

**Fig. S6.** Comparisons of the tools on the six datasets containing various bicluster patterns: trend-preserving, column-constant, shift-scale, shift, scale and constant-upregulated patterns in terms of Relevance scores.

**
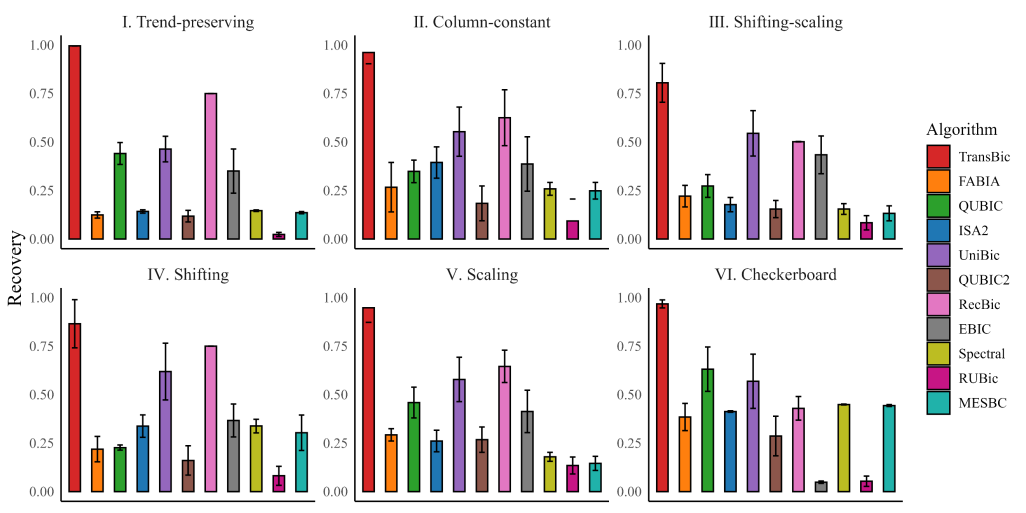
**

**Fig. S5.** Comparisons of the tools on the six datasets containing various bicluster patterns: trend-preserving, column-constant, shift-scale, shift, scale and constant-upregulated patterns in terms of Recovery scores.

**
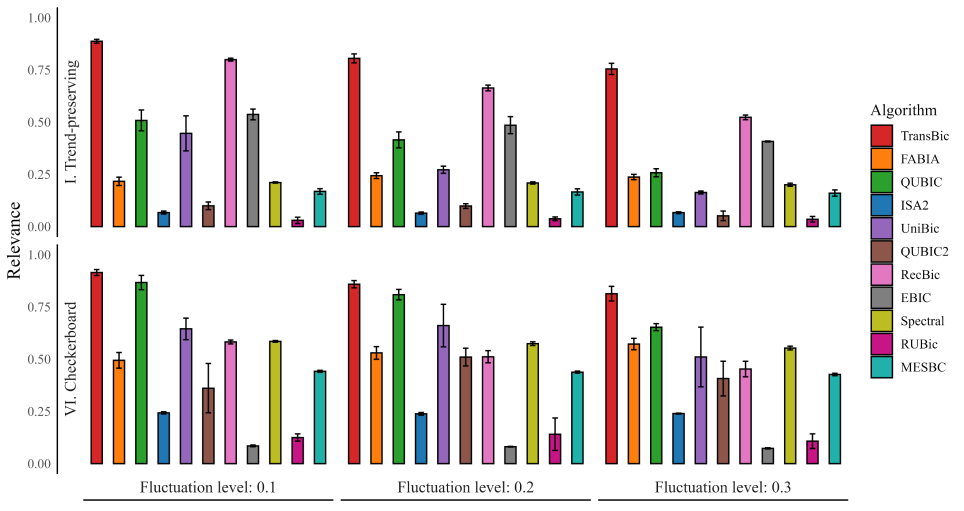
**

**Fig. S9.** Comparisons of the tools on datasets with different fluctuation levels: 0.1, 0.2 and 0.3 for trend-preserving and constant-upregulated biclusters respectively in terms of Relevance scores.

**
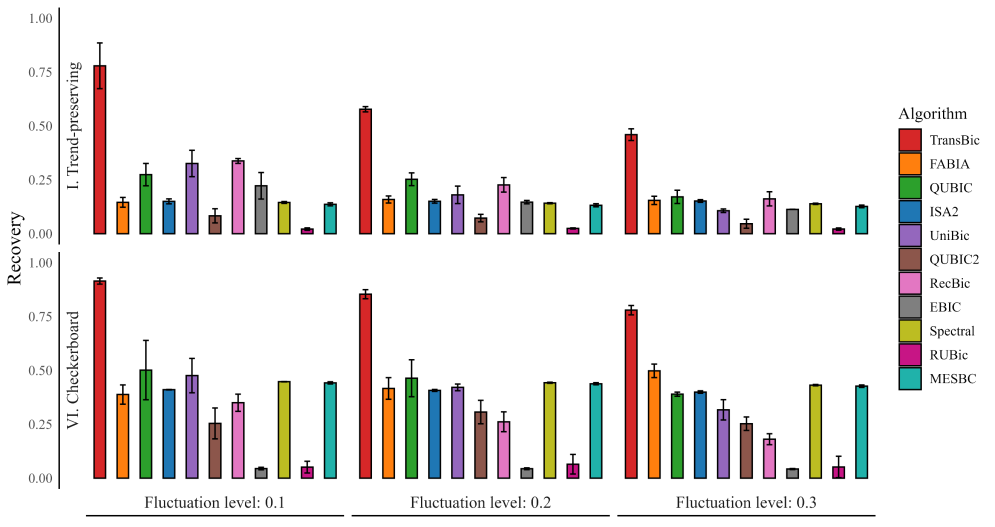
**

**Fig. S8.** Comparisons of the tools on datasets with different fluctuation levels: 0.1, 0.2 and 0.3 for trend-preserving and constant-upregulated biclusters respectively in terms of Recovery scores.

**
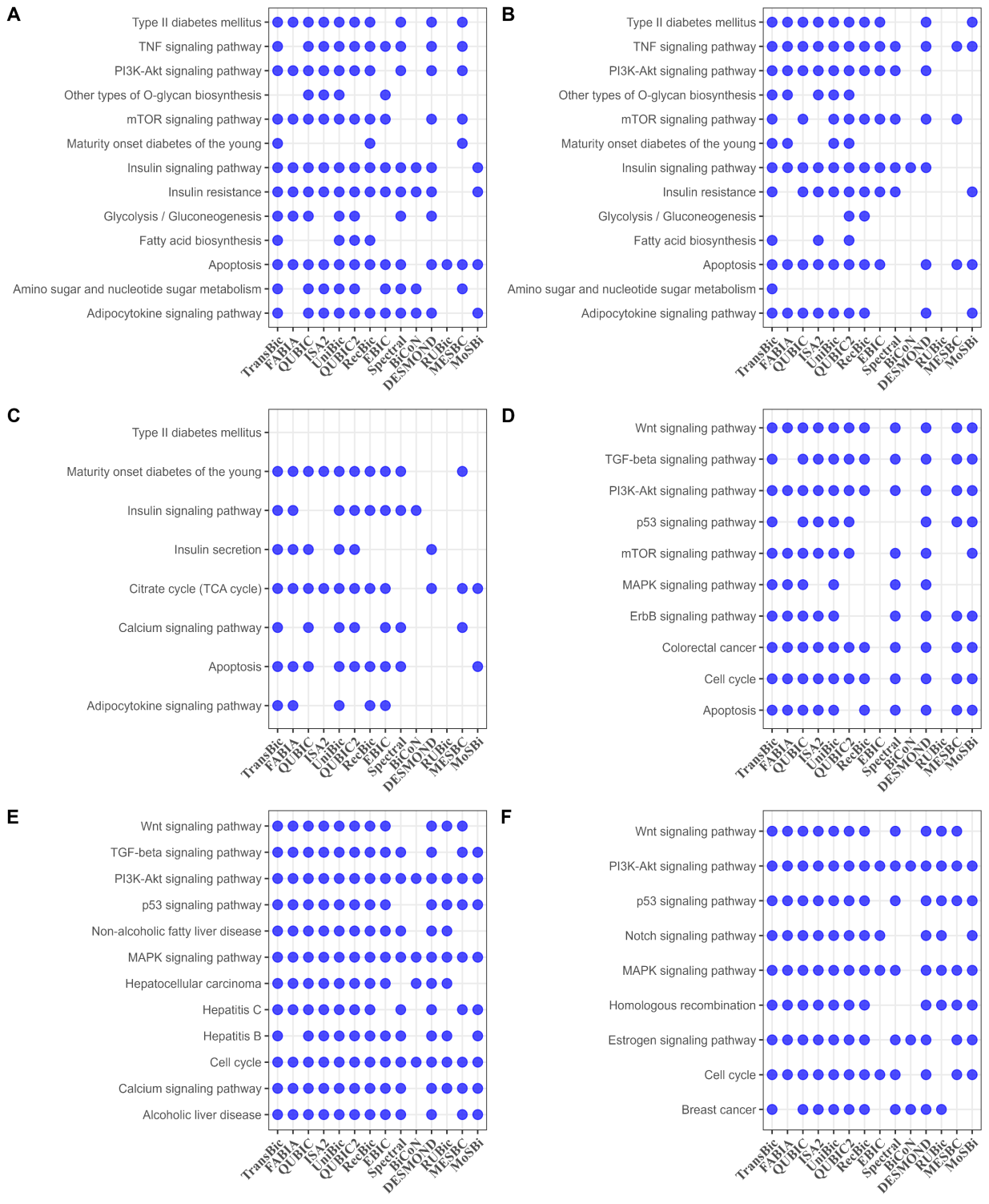
**

**Fig.S10.** Enrichment results of the tools on the related KEGG pathways with corresponding diseases. **A**-**C**. Enrichment results of the tools on the related KEGG pathways with type 2 diabetes at the three different tissues: adipose, liver and pancreas. **D**. Enrichment results of the tools on the related pathways with colorectal cancer. **E**. Enrichment results of the tools on the related pathways with hepatocellular carcinoma. **F**. Enrichment results of the tools on the related pathways with breast cancer.


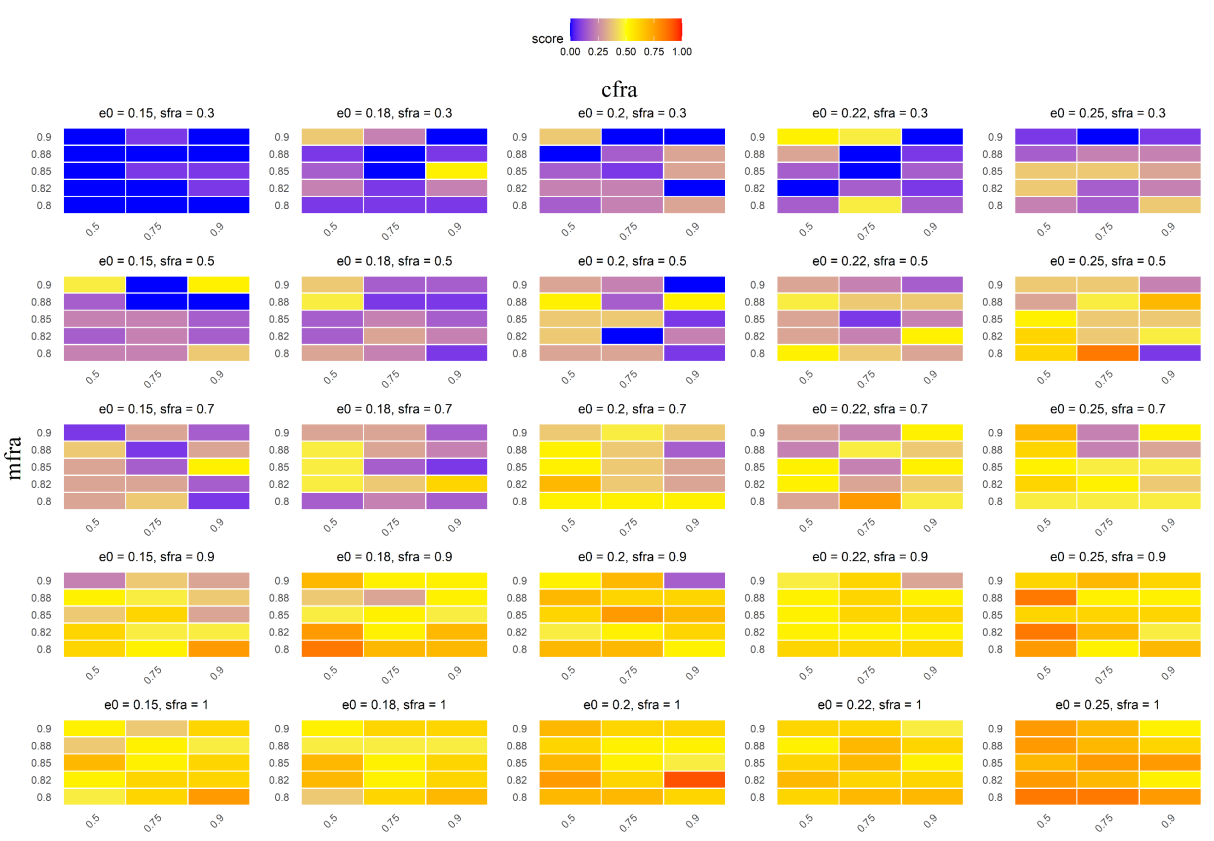


**Fig.S11.** Performance scores of TransBic on the T2D (adipose) dataset under different combinations of parameters: $e_{0}$, sfra, mfra ($\gamma_{0}$), cfra ($\sigma$).


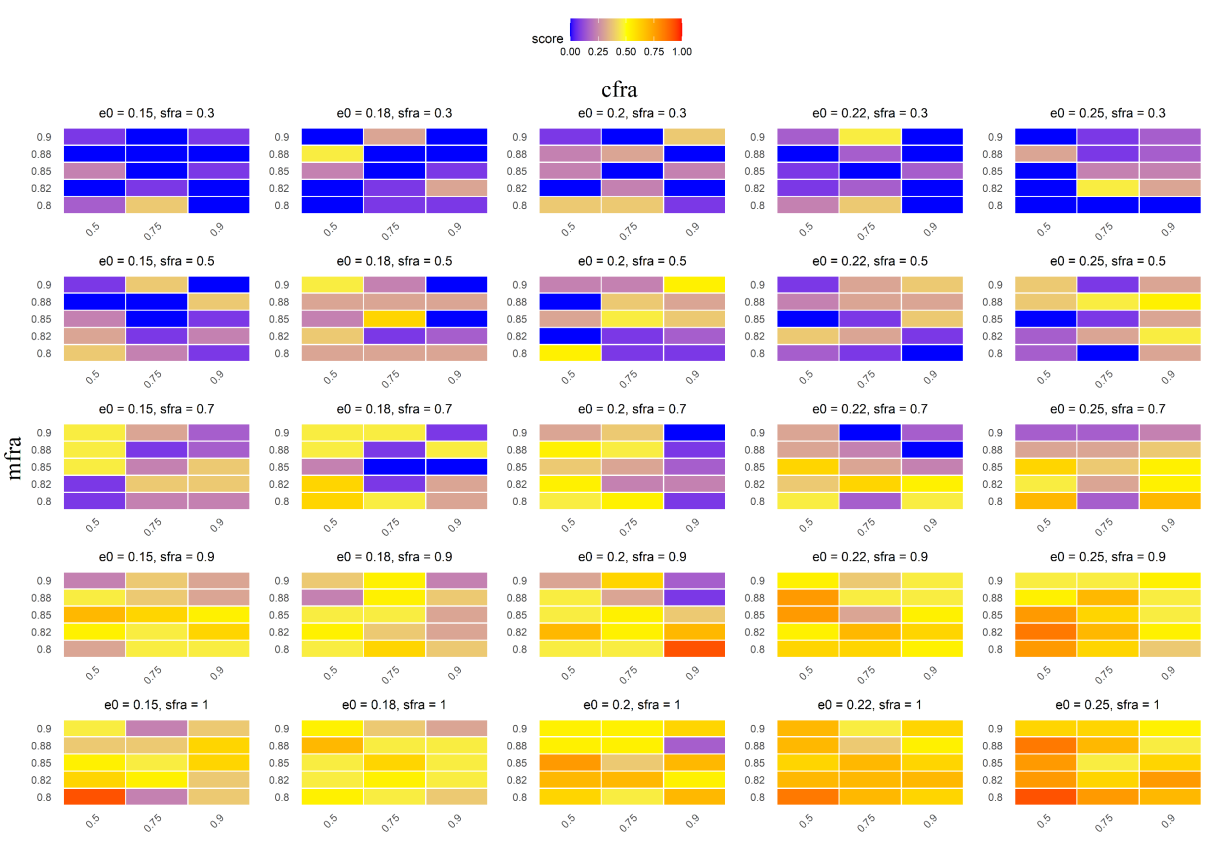


**Fig.S12.** Performance scores of TransBic on the T2D (liver) dataset under different combinations of parameters: $e_{0}$, sfra, mfra ($\gamma_{0}$), cfra ($\sigma$).


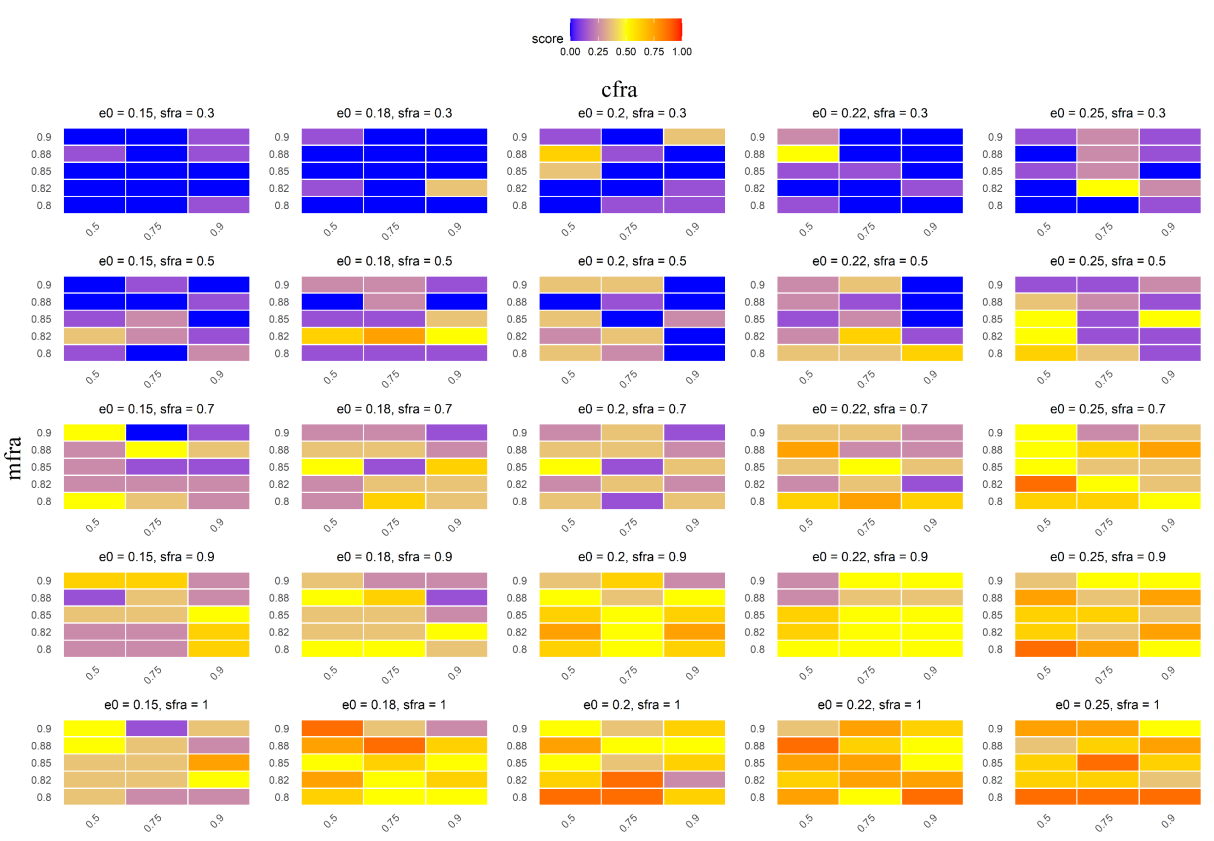


**Fig.S13.** Performance scores of TransBic on the T2D (pancreas) dataset under different combinations of parameters: $e_{0}$, sfra, mfra ($\gamma_{0}$), cfra ($\sigma$).


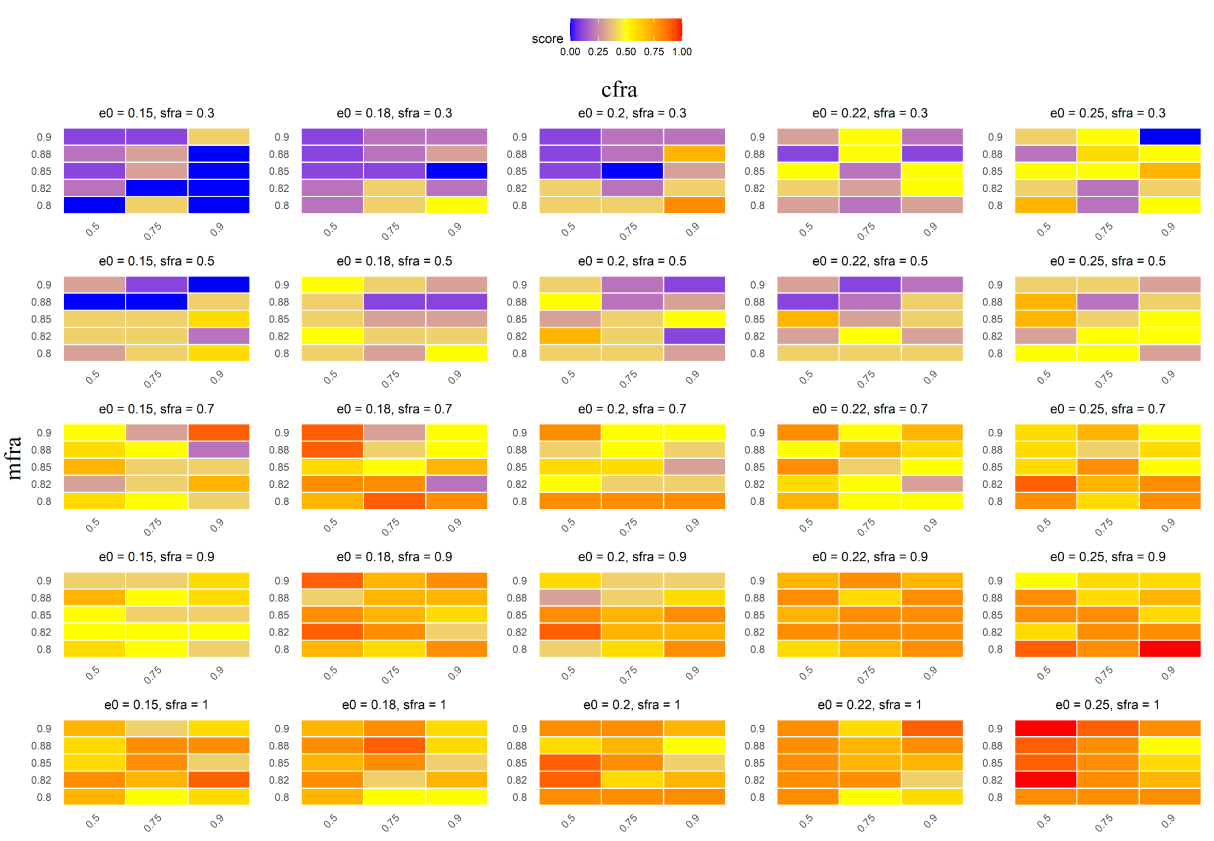


**Fig.S14.** Performance scores of TransBic on the CC dataset under different combinations of parameters: $e_{0}$, sfra, mfra ($\gamma_{0}$), cfra ($\sigma$).


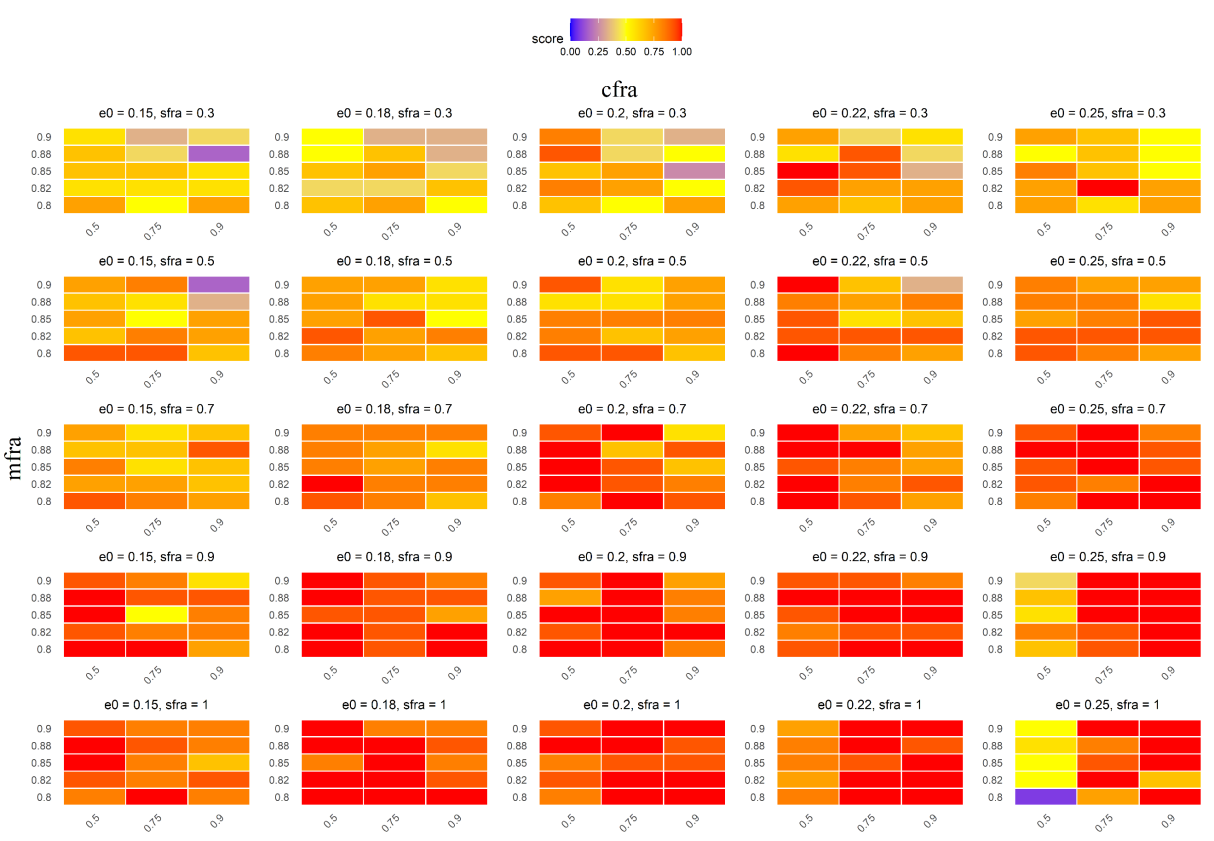


**Fig.S15.** Performance scores of TransBic on the HC dataset under different combinations of parameters: $e_{0}$, sfra, mfra ($\gamma_{0}$), cfra ($\sigma$).


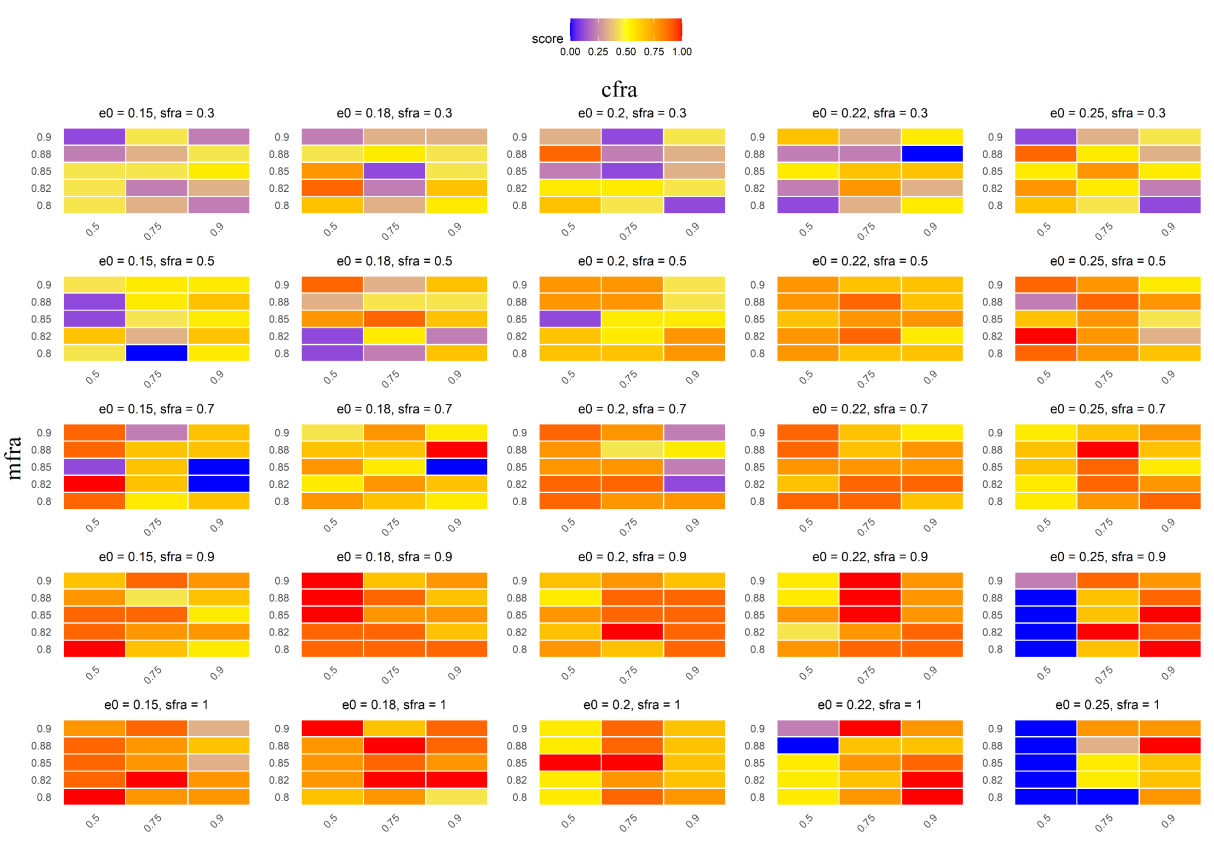


**Fig.S16.** Performance scores of TransBic on the BC dataset under different combinations of parameters: $e_{0}$, sfra, mfra ($\gamma_{0}$), cfra ($\sigma$).

Supplementary Note:

Comparison of all the algorithms in identifying BTP-biclusters

Consider a bicluster $\left( I, J \right)$ with a BTP pattern in partition $J_{1},J_{2}, J_{3}, \cdots, J_{l}$, where $I=I_{1}\cup I_{2}$ and $J=\bigcup_{i=1}^{l} J_{i}$. The gene set *I* and condition set $J$ were randomly selected, and the entries were rearranged so that for any gene ${g_{i}\in I}_{1}$, $\max\left\{ a_{i,j}:c_{j}\in J_{d} \right\}<\min\left\{ a_{i,j}:c_{j}\in J_{d+1} \right\},$ and for any gene $\in I_{2}$, $\min\left\{ a_{i,j}:c_{j}\in J_{d} \right\}>\max\left\{ a_{i,j}:c_{j}\in J_{d+1} \right\}$, where $d=1,2,\ldots,l-1$. The size of the BTP-bicluster is given by $\left( \left| I_{1} \right|,\left| I_{2} \right| \right)\times\left( \left| J_{1} \right|/m,\left| J_{2} \right|/m,\left| J_{3} \right|/m,\cdots,\left| J_{l} \right|/m \right)$.

To compare all algorithms in the first two scenarios, we generated 21 expression matrices with 500, 1000, and 2000 rows and 20, 30, …, and 80 columns. For each expression matrix, we implanted 5 biclusters with their respective sizes: (40, 40) × (1/10, 1/10, 1/10), (100, 50) × (1/5, 1/5), (40, 80) × (1/5, 2/5), (60, 0) × (1/6, 1/6, 2/6) and (50, 0) × (1/5, 1/5, 1/5, 1/5). For the seven datasets with the same number of rows, we recorded their Performance scores in Fig. 3A. We further expanded these results for different numbers of columns in Fig.3B. Comparisons based on Recovery and Relevance metrics are illustrated in Figs. S2A-S2B and S3A-S3B, respectively.

For overlapping biclusters, we generated four groups of synthetic datasets corresponding to different overlapping levels of biclusters. Specifically, we first generated background matrices with 1000 rows and 50 columns, and then implanted three different biclusters with their respective sizes: (100, 50) × (1/3, 1/3), (200, 100) × (1/6, 1/6, 1/6) and (150, 0) × (1/2, 1/6) in each matrix. Let $\omega$ be an integer representing the number of overlapping genes. The first two biclusters have $\omega$ genes in common and the last two biclusters have another $\omega$ genes in common ($\omega$ = 0, 20, 40 and 60). Overlap ratios are calculated by dividing the number of shared genes by the total gene count in each bicluster. The maximum overlapping ratio for all biclusters is 0, 0.2, 0.4, and 0.6, respectively. We generated four datasets per group. Comparative results using Performance, Recovery, and Relevance metrics are shown in Fig. 3C, Fig. S2C, and Fig. S3C.

To test the robustness of TransBic, we compared it with other biclustering methods using datasets containing noisy BTP-biclusters. Background matrices were created with 1000 genes and 50 conditions, and three biclusters were implanted with sizes: (40, 40) × (1/5, 1/10, 1/5), (100, 50) × (1/5, 2/5), and (60, 0) × (1/10, 1/10, 1/10). Noise was added to the BTP-biclusters at four levels: 0, 0.1, 0.2, and 0.3. Five datasets were generated per noise level. Results comparing Performance, Recovery, and Relevance metrics are presented in Fig. 3D, Fig. S2D, and Fig. S3D.

Finally, we evaluated TransBic and other algorithms using data that mimics real expression datasets. The background matrix contains 10,000 genes and 50 conditions and considering GO terms and KEGG pathway sizes, which range from tens to thousands. We implanted five BTP-biclusters with four different sizes: (400, 800) × (1/5, 2/5), (400, 400) × (1/10, 1/10, 1/10), (100, 0) × (1/4, 1/4, 1/10), and (50, 0) × (1/5, 1/5, 1/5, 1/5). Four datasets were generated for each case. Comparative results using Performance, Recovery, and Relevance metrics are shown in Fig. 3E, Fig. S2E, and Fig. S3E. mimicking real expression data.

Comparison of all the algorithms in identifying different patterns, especially against data fluctuations

Since the BTP-pattern is a generalization of all the previously mentioned patterns, except for the row-constant pattern, TransBic is capable of identifying biclusters with the following patterns: column-constant, shifting-scaling, shifting, scaling, checkerboard, order-preserving, and trend-preserving. The checkerboard pattern is a specific case of the BTP-pattern, characterized by two buckets, and can be detected by extracting the upregulated or downregulated portions within the BTP-bicluster. In the following, we evaluated the performance of all algorithms in identifying biclusters exhibiting these patterns, excluding the row-constant pattern. We generated background matrices mimicking real gene expression datasets and implanted four biclusters of varying sizes (row numbers × column numbers): 1200$\times$15, 800$\times$10, 100$\times$20, and 50$\times$15. We did not separately analyze order-preserving biclusters here, as they inherently represent the same underlying pattern as trend-preserving patterns. For each pattern, four datasets were generated through repetition. Since data fluctuations are disregarded in this analysis, we set the minimum number of elements in each bucket for TransBic to 1, except for the checkerboard pattern. To compare all algorithms simultaneously on both Relevance and Recovery scores, we selected the highest-performing result for each algorithm based on the best Performance score and displayed the corresponding Relevance and Recovery scores in Fig. 4A. The Performance, Recovery and Relevance metrics are recorded in Figs. S4-6.

We further evaluated all algorithms under varying levels of data fluctuations. Given that column-constant, shifting, scaling, and shifting-scaling patterns are all classified as trend-preserving, we employed datasets containing both trend-preserving and checkerboard biclusters to assess algorithmic performance across different fluctuation levels. As with previous analyses, we selected the highest-performing result for each algorithm based on the best Performance score, with the corresponding Relevance and Recovery scores presented in Fig. 4B. The Performance, Recovery and Relevance metrics are recorded in Figs. S7-9.

Theoretical basis for defining $\boldsymbol{S}$

The foundation for defining$S$is based on the observation that any pair of acyclic tournament digraphs in$S$tend to exhibit more common arcs than other digraph pairs, and vice versa. The observation informs the initialization of $S$ with the pair of digraphs sharing the most arcs and the subsequent expansion of $S$ by iteratively incorporating a new digraph if it shares the most arcs with a digraph in$S$. However, this is not sufficient to guarantee that the new digraph shares a significant CAMT subdigraph with$S$. For example, there may exist another set $S^{'}$corresponding to a distinct CAMT subdigraph where$S\bigcap S^{'}\neq\emptyset$. If a new digraph $D_{\alpha}$shares the most arcs with a digraph $D_{\beta}\in S\bigcap S^{'}$, then according to the observation above, $D_{\alpha}$may share a significant CMAT subdigraph with$S^{'}$rather than with$S$. To prevent the inclusion of such digraphs during the expansion of$S$, which could lead to a deviation in$H$, we introduce an additional criterion. The new digraph$D_{\alpha}$is expected to contain as many arcs from $H$ as possible, particularly in contrast to the set$L$of low-frequency arcs, both$H$and$L$originating from the previous iteration. Under the aforementioned criterion, this can be quantified using the Kullback-Leibler divergence score [9] between$H$and$L$for$D_{\alpha}$.

Computation for $\boldsymbol{p}$ to examine a candidate CMAT subdigraph.

Before outputting, the candidate CMAT subdigraph must be checked for statistical significance. The details are as follows:

For simplicity, assume that in an expression matrix $\boldsymbol{A}_{n\times m}$​, each gene $g_{i}, (i=1,2,\ldots,n)$ corresponds to a single acyclic tournament digraph $D_{i_{-1}}$​. For $\boldsymbol{A}_{n\times m}$​, there are $m!$ possible permutations for each row vector, and correspondingly, there are $m!$ possible acyclic tournament digraphs. Each of these digraphs has a probability of occurrence equal to $1/{m!}$. Assume there are $c$ possible permutations that contain the CMAT subdigraph while allowing a maximum fraction $e_{0}$​ of missing arcs. Each digraph then has an average probability $p_{r}=c/{m!}$ of containing the CMAT subdigraph. All digraphs derived from $\boldsymbol{A}_{n\times m}$ are assumed to be independent of each other, and thus the number of conforming digraphs follows a binomial distribution. This allows us to compute the probability of various numbers of digraphs containing the CMAT subdigraph. However, since $m!$ is extremely large, calculating $p_{r}$ by enumerating all possibilities and checking for compliance is computationally impractical. Therefore, a simplified approach is proposed:

Let $A_{T}$​ be the total number of arcs in the CMAT, and $A$ be the number of intersected arcs between an acyclic tournament digraph and the CMAT. A digraph is considered to contain the CMAT subdigraph if $1-A/{A_{T}}<e_{0}$​. In this formula, $e_{0}$​ and $A_{T}$*​*​ are known, and we need to list all possible counts of $A$ and compute their respective probabilities of occurrence.

For all partite sets $P_{1}$, $P_{2}$, $\cdots$, $P_{l}$, let $A_{P_{1},P_{2}}$ denotes the number of arcs in a digraph that directed from $P_{2}$ to $P_{1}$. The possible values of $A_{P_{1},P_{2}}$ range from 0 to $\left| P_{1} \right|*\left| P_{2} \right|$, where $\left| P_{1} \right|$ and $\left| P_{2} \right|$ denote the number of elements in partite set $P_{1}$ and $P_{2}$, respectively. Next, we compute the probabilities of all possible values of $A_{P_{1},P_{2}}$. This can be formulated as a classical permutation and combination problem: distributing $l$ identical apples into $k$ identical dishes, where dishes can be empty, and the number of apples in each dish cannot exceed $r$. By setting *l* as $A_{P_{1},P_{2}}$, *k* as $\left| P_{2} \right|$ and *r* as $\left| P_{1} \right|$. We traverse all possible values of $A_{P_{1},P_{2}}$, and their corresponding probabilities are given by the ratio of the number of ways each value can occur to the total number of possible distributions.

After merging partite sets *P*_1_ and *P*_2_ into a single set {*P*_1,_ *P*_2_}, we compute the probabilities of all possible values of $A_{\left\{ P_{1},P_{2} \right\},P_{3}}$ using the same approach. This procedure is repeated, allowing us to obtain the probabilities of all possible values of $A_{P_{1},P_{2}}$, $A_{\left\{ P_{1},P_{2} \right\},P_{3}}$, $A_{\left\{ P_{1},P_{2},P_{3} \right\},P_{4}}$, $\cdots$, $A_{\left\{ P_{1},\cdots,P_{l-1} \right\},P_{l}}$. The value of $A$ is the sum of $A_{P_{1},P_{2}}$*,* $A_{\left\{ P_{1},P_{2} \right\},P_{3}}$*,* $A_{\left\{ P_{1},P_{2},P_{3} \right\},P_{4}}$*,* $\cdots$*,* $A_{\left\{ P_{1},\cdots,P_{l-1} \right\},P_{l}}$, and its corresponding probability is the product of their individual probabilities. Probabilities corresponding to the same value of $A$ need to be summed. Consequently, the probability $p_{r}$ can be computed as

$$\begin{aligned} p_{r}&\mathbb{=P}\left( 1-\frac{A}{A_{T}}<e_{0} \right) \\ &=\mathbb{p}\left( A>A_{T}*\left( 1-e_{0} \right) \right) \\ &=\mathbb{p}\left( \left\lceil A_{T}*\left( 1-e_{0} \right) \right\rceil\right)+\mathbb{p}\left( \left\lceil A_{T}*\left( 1-e_{0} \right) \right\rceil+1 \right)+\mathbb{p}\left( \left\lceil A_{T}*\left( 1-e_{0} \right) \right\rceil+2 \right)+ \\ & \mathbb{p}\left( \left\lceil A_{T}*\left( 1-e_{0} \right) \right\rceil+3 \right) \mathbb{+\cdots+P}\left( A_{T} \right)\# = 1 \backslash* GB2 ⑴ \end{aligned}$$

Suppose $t$ digraphs containing the CMAT subdigraph, the *p*-value $p$ could be calculated as

$$\begin{aligned} p=C_{n}^{t}*{p_{r}}^{t}*\left( 1-p_{r} \right)^{n-t}+C_{n}^{t+1}*{p_{r}}^{t+1}*\left( 1-p_{r} \right)^{n-\left( t+1 \right)}+C_{n}^{t+2}*{p_{r}}^{t+2}*\left( 1-p_{r} \right)^{n-\left( t+2 \right)}+ \\ C_{n}^{t+3}*{p_{r}}^{t+3}*\left( 1-p_{r} \right)^{n-\left( t+3 \right)}+ \cdots+C_{n}^{n}*{p_{r}}^{n}\# = 2 \backslash* GB2 ⑵ \end{aligned}$$

Given a threshold $\alpha$, we consider the CMAT subdigraph is of significance if $p<\alpha.$

Note that each gene $g_{i}, (i=1,2,\ldots,n)$ also corresponds to a acyclic tournament digraph $D_{i_{-2}}$​. Thus, the formula ⑴ is changed to

$$\begin{aligned} p_{r}&\mathbb{=P}\left( 1-\frac{A}{A_{T}}<e_{0} \right)+\mathbb{p}\left( 1-\frac{A}{A_{T}}>1-e_{0} \right) \\ &=\mathbb{p}\left( A>A_{T}*\left( 1-e_{0} \right) \right)+\mathbb{p}\left( A<A_{T}*e_{0} \right) \\ &=\mathbb{p}\left( \left\lceil A_{T}*\left( 1-e_{0} \right) \right\rceil\right)+\mathbb{p}\left( \left\lceil A_{T}*\left( 1-e_{0} \right) \right\rceil+1 \right)+\mathbb{p}\left( \left\lceil A_{T}*\left( 1-e_{0} \right) \right\rceil+2 \right)+ \\ & \mathbb{p}\left( \left\lceil A_{T}*\left( 1-e_{0} \right) \right\rceil+3 \right) \mathbb{+\cdots+P}\left( A_{T} \right)+0+1+2+3+\cdots+\mathbb{p}\left( \left\lfloor A_{T}*e_{0} \right\rfloor\right)\# = 3 \backslash* GB2 ⑶ \end{aligned}$$

Tool Selection and Implementation for Comparison

To demonstrate the superiority of TransBic in biclustering expression data, we benchmarked TransBic against thirteen state-of-the-art biclustering tools, including QUBIC [10], UniBic [11], QUBIC2 [12], EBIC [13], RecBic [14], FABIA [15], Spectral [16], ISA [17], DESMOND [18], BiCoN [19], RUBic [20], MESBC [21], and MoSBi [22], using both simulated and real expression datasets. These tools encompass all established trend-preserving biclustering methods. ARBic [23] was excluded from the analysis as it defaults to RecBic when the number of columns is below 500, a threshold sufficient for our experimental design. Additionally, we incorporated recent high-impact biclustering tools published in the past five years—QUBIC2, DESMOND, BiCoN, RUBic, MESBC, and MoSBi—alongside classic algorithms such as Spectral, QUBIC, ISA, and FABIA, the latter three having been identified as top performers for predicting FGMs by Saelens, Cannoodt [24]. We executed ISA, FABIA, QUBIC, UniBic, Spectral, MESBC, and MoSBi using their respective R packages in version 4.1.2, while QUBIC2, EBIC, RecBic, and RUBic were run by compiling source code on Ubuntu 18.04 (QUBIC2, RecBic, and RUBic) and Windows 10 (EBIC). DESMOND and BiCoN were implemented in Python 3.7. Given that DESMOND and BiCoN require biological networks as input, we compared TransBic with the remaining eight biclustering algorithms for their ability to detect biclusters with different patterns in simulated datasets. For real datasets, we evaluated all algorithms for their effectiveness in identifying biologically significant FGMs, specifically focusing on disease expression data to identify KEGG pathways associated with specific diseases.

GO and KEGG enrichment for the output biclusters

We firstly filtered biclusters by excluding biclusters with fewer than five genes or conditions and removing highly overlapping biclusters (Jaccard index > 0.7), following the guidelines of Saelens, Cannoodt [24]. Then we performed GO and KEGG analyses for the remaining biclusters. The DAVID database [25, 26] was used to transform other gene identifies into Entrez Gene IDs, and the R package Clusterprofiler [27, 28] facilitated the enrichment analysis. In ClusterProfiler, genes associated with probes in each identified bicluster were used as the test set, while all genes associated with probes in the input dataset formed the gene universe. Multiple testing correction was performed using the Benjamini-Hochberg method [29], with a p-value cutoff of 0.05 and a q-value cutoff of 0.2. For each tool, we selected its optimal result to compare, which has the lowest average p-value after maximizing enrichment rates across all relevant KEGG pathways. For BiCoN and DESMOND, we downloaded the Protein-Protein Interaction Networks (PPI) from BioGRID in *Mus musculus* (version 4.4.233) and the PPI provided by BiCoN in *Homo sapiens*. Gene identifiers were mapped to Entrez Gene IDs and filtered in their respective ways. We selected disease pathways and their related pathways from the KEGG database as benchmarks to compare all algorithms (see Table S4 for details). We evaluated these algorithms based on their enrichment performances in these pathways.

**Spearman's rank correlation coefficient between bucket trends and age, diet**

For the T2D expression datasets, each sample is assigned two distinct labels: age (“Week 1”, “Week 9”, or “Week 18”) and diet (“Regular Chow” or “High-Fat Diet”). These labels represent two independent factors: the development of age and the variation in dietary conditions. We further define a vector to represent the progression of age, where each sample is assigned a value based on its time point:

0 for samples with “Week 1”

1 for samples with “Week 9”

2 for samples with “Week 18”

Similarly, to represent the variation of diet, we define another vector where each sample is assigned a value based on its dietary condition:

0 for samples with “Regular Chow”

1 for samples with “High-Fat Diet”

For each BTP-pattern, a vector can also be defined to represent the order of its buckets. All samples in the first bucket are assigned a value of 0, with subsequent buckets incrementing sequentially (e.g., 1, 2, etc.).

The correlation between a BTP-pattern and age can be calculated using Spearman's rank correlation coefficient [30] between the defined vector for the BTP-pattern and age. Similarly, the correlation between a pattern and diet is computed as the Spearman correlation coefficient between the defined vector for the BTP-pattern and diet. The Spearman correlation can be calculated using the “cor” function in R.

Complexity estimation

Given the expression matrix $\boldsymbol{A}_{\boldsymbol{n}\boldsymbol{\times}\boldsymbol{m}}$ with $\boldsymbol{n}$ genes and $\boldsymbol{m}$ conditions, Step 1 constructs $\mathbf{2}\boldsymbol{n}$ acyclic tournament digraphs. These digraphs are stored in a matrix of size $\mathbf{2}\boldsymbol{n}\boldsymbol{\times}\boldsymbol{m}\mathbf{(}\boldsymbol{m}\mathbf{-}\mathbf{1}\mathbf{)}$, resulting in a computational complexity of $\boldsymbol{O}\left( \boldsymbol{n}\boldsymbol{m}^{\mathbf{2}} \right)$. Step 2 involves two main computationally-intensive parts: the initialization of $\boldsymbol{S}$ and the subsequent growth. In order to initialize $\boldsymbol{S}$ using the largest pair of digraphs, we enumerate all pairs of digraphs and calculate the number of their common arcs, which takes at most $\boldsymbol{O}\left( \boldsymbol{n}^{\mathbf{2}}\boldsymbol{m}^{\mathbf{2}} \right)$. Assuming that each bicluster has a maximum gene size of $\boldsymbol{\omega}$, the number of digraphs in $\boldsymbol{S}$ will not exceed $\boldsymbol{\omega}$ correspondingly. Thus, traversing over $\boldsymbol{2}\boldsymbol{n}$ digraphs to add additional digraphs into $\boldsymbol{S}$ takes a total of $\boldsymbol{O}\left( \boldsymbol{\omega}^{\mathbf{2}}\boldsymbol{n}\boldsymbol{m}^{\mathbf{2}} \right)$. In Step 3, detecting a CMAT subdigraph runs in $\boldsymbol{O}\left( \boldsymbol{m}^{\mathbf{5}} \right)$, and in Step 4, computing p-value for a CMAT subdigraph runs at $\boldsymbol{O}\left( \boldsymbol{m}^{\mathbf{6}} \right)$ in the worst case. If the final output consists of $\boldsymbol{o}$ biclusters, the overall time complexity of TransBic is the maximum of the following: $\boldsymbol{O}\left( \boldsymbol{n}^{\mathbf{2}}\boldsymbol{m}^{\mathbf{2}} \right)$, $\boldsymbol{O}\left( \boldsymbol{o}\boldsymbol{\omega}^{\mathbf{2}}\boldsymbol{n}\boldsymbol{m}^{\mathbf{2}} \right)$, $\boldsymbol{O}\left( \boldsymbol{om}^{\mathbf{6}} \right)$. The running time of TransBic scales linearly with the number of rows and columns in the data matrix, making it a practical algorithm for identifying BTP-biclusters from gene expression data (see Table S5-S6 for the comparison of running time of the tools).

Impact of parameters on TransBic in real expression datasets

Our proposed algorithm, TranBic, consists of four main parameters: $e_{0}$, $\gamma_{0}$, $\sigma$, and $sfra$, all of which are threshold parameters. In our code, $\gamma_{0}$ and $\sigma$ are represented as $mfra$ and $cfra$, respectively. The parameter $sfra$ is derived in Step 2. Once the algorithm converges to a stable set $H$, which is arcs of the high-frequency digraph $D$, we further refine $H$ by retaining only the proportion $sfra$ of arcs corresponding to the highest proportion $sfra$ of frequency values in the rows in $S$. This parameter, $sfra$, regulates the arcs of the resulting high-frequency digraph $D$, thereby determining the search space for the following CMAT subdigraphs. Parameters $mfra$ and $cfra$ are derived in Step 3 and serve as key threshold parameters for the refinement of the identified CMAT subdigraph (BTP-pattern) (see Step 3 for details). Finally, $e_{0}$ acts as a noise threshold parameter, allowing for the identification of genes within the permitted $e_{0}$ error rate that align with the desired BTP-pattern. For each of these four parameters, we selected multiple values around their default settings and evaluated their metric scores on real datasets (see the parameter settings in Supplementary Table 1). As shown in Figures S11-S16, TransBic with $sfra=$0.9, 1.0 perform better across all datasets. $sfra=$0.9, 1.0 indicates that no filtering is applied to the stable $H$ obtained from Step 2, demonstrating that the arcs $H$ identified in Step 2 can accurately delineate the contours of the CMAT subdigraphs. For the parameter $e_{0}$, $e_{0}=0.25$ typically yields the best performance in the first four datasets (T2D: adipose, liver, pancreas) and CC. $e_{0}=$ 0.2 and 0.22 are optimal for HC, while in the BC dataset, $e_{0}=0.18$ shows superior results. These findings indicate that $e_{0}$ is a noise parameter that depends on the specific dataset. In contract, for $mfra$ and $cfra$, we did not observe consistently advantageous ranges across the datasets; rather, it appears that the combination of $mfra$ and $cfra$ may yield optimal results in certain datasets.

1. Utzschneider, K.M., et al., *Impact of intra-abdominal fat and age on insulin sensitivity and β-cell function.* Diabetes, 2004. **53**(11): p. 2867-2872.

2. Vieira-Lara, M.A., et al., *Age and diet modulate the insulin-sensitizing effects of exercise: a tracer-based oral glucose tolerance test.* Diabetes, 2023: p. db220746.

3. López-Otín, C., et al., *Hallmarks of aging: An expanding universe.* Cell, 2023. **186**(2): p. 243-278.

4. Yida, Z., et al., *High fat diet-induced inflammation and oxidative stress are attenuated by N-acetylneuraminic acid in rats.* Journal of biomedical science, 2015. **22**: p. 1-10.

5. Lee, Y.S., et al., *Inflammation is necessary for long-term but not short-term high-fat diet–induced insulin resistance.* Diabetes, 2011. **60**(10): p. 2474-2483.

6. Miotto, P.M., P.J. LeBlanc, and G.P. Holloway, *High-fat diet causes mitochondrial dysfunction as a result of impaired ADP sensitivity.* Diabetes, 2018. **67**(11): p. 2199-2205.

7. Mukherjee, A., et al., *Type 2 diabetes as a protein misfolding disease.* Trends in molecular medicine, 2015. **21**(7): p. 439-449.

8. Anhê, F.F., et al., *Type 2 diabetes influences bacterial tissue compartmentalisation in human obesity.* Nature Metabolism, 2020. **2**(3): p. 233-242.

9. Kullback, S. and R.A. Leibler, *On information and sufficiency.* The annals of mathematical statistics, 1951. **22**(1): p. 79-86.

10. Li, G., et al., *QUBIC: a qualitative biclustering algorithm for analyses of gene expression data.* Nucleic acids research, 2009. **37**(15): p. e101-e101.

11. Wang, Z., et al., *UniBic: Sequential row-based biclustering algorithm for analysis of gene expression data.* Scientific reports, 2016. **6**(1): p. 1-10.

12. Xie, J., et al., *QUBIC2: a novel and robust biclustering algorithm for analyses and interpretation of large-scale RNA-Seq data.* Bioinformatics, 2020. **36**(4): p. 1143-1149.

13. Orzechowski, P., et al., *EBIC: an evolutionary-based parallel biclustering algorithm for pattern discovery.* Bioinformatics, 2018. **34**(21): p. 3719-3726.

14. Liu, X., et al., *RecBic: a fast and accurate algorithm recognizing trend-preserving biclusters.* Bioinformatics, 2020. **36**(20): p. 5054-5060.

15. Hochreiter, S., et al., *FABIA: factor analysis for bicluster acquisition.* Bioinformatics, 2010. **26**(12): p. 1520-1527.

16. Kluger, Y., et al., *Spectral biclustering of microarray data: coclustering genes and conditions.* Genome research, 2003. **13**(4): p. 703-716.

17. Bergmann, S., J. Ihmels, and N. Barkai, *Iterative signature algorithm for the analysis of large-scale gene expression data.* Physical review E, 2003. **67**(3): p. 031902.

18. Zolotareva, O., et al., *Identification of differentially expressed gene modules in heterogeneous diseases.* Bioinformatics, 2021. **37**(12): p. 1691-1698.

19. Lazareva, O., et al., *BiCoN: network-constrained biclustering of patients and omics data.* Bioinformatics, 2021. **37**(16): p. 2398-2404.

20. Sriwastava, B.K., et al., *RUBic: rapid unsupervised biclustering.* BMC bioinformatics, 2023. **24**(1): p. 435.

21. Liu, F., et al., *MESBC: A novel mutually exclusive spectral biclustering method for cancer subtyping.* Computational Biology and Chemistry, 2024. **109**: p. 108009.

22. Rose, T.D., et al., *MoSBi: Automated signature mining for molecular stratification and subtyping.* Proceedings of the National Academy of Sciences, 2022. **119**(16): p. e2118210119.

23. Liu, X., et al., *ARBic: an all-round biclustering algorithm for analyzing gene expression data.* NAR Genomics and Bioinformatics, 2023. **5**(1): p. lqad009.

24. Saelens, W., R. Cannoodt, and Y. Saeys, *A comprehensive evaluation of module detection methods for gene expression data.* Nature communications, 2018. **9**(1): p. 1090.

25. Huang, D.W., B.T. Sherman, and R.A. Lempicki, *Systematic and integrative analysis of large gene lists using DAVID bioinformatics resources.* Nature protocols, 2009. **4**(1): p. 44-57.

26. Sherman, B.T., et al., *DAVID: a web server for functional enrichment analysis and functional annotation of gene lists (2021 update).* Nucleic Acids Res, 2022. **10**.

27. Wu, T., et al., *clusterProfiler 4.0: A universal enrichment tool for interpreting omics data.* The Innovation, 2021. **2**(3): p. 100141.

28. Yu, G., et al., *clusterProfiler: an R package for comparing biological themes among gene clusters.* Omics: a journal of integrative biology, 2012. **16**(5): p. 284-287.

29. Hochberg, Y. and Y. Benjamini, *More powerful procedures for multiple significance testing.* Statistics in medicine, 1990. **9**(7): p. 811-818.

30. Spearman, C., *The proof and measurement of association between two things, Amer. J. Psychol., 15, 88.(1906).* A footrule for measuring correlation,” Brit. Jour. Psychol, 1904. **2**: p. 89.
