# Supplementary material for: TransBic: bucket trend-preserving biclustering for finding local and interpretable expression patterns
Source: Brief Bioinform. 2025 Feb 5;26(1):bbaf050. doi: 10.1093/bib/bbaf050 (PMC11794469; doi:10.1093/bib/bbaf050)
Supplement: Supplementary_Table_1_bbaf050 [file supplementary_table_1_bbaf050.pdf]

Parameters values		
**Methods**	**Parameter values tested for Synthetic Data**	**Parameter values tested for Real Data**
QUBIC	r={1,2,3}<br>q={0.05,0.06, 0.07, 0.08, 0.09, 0.1, 0.25, 0.35, 0.5}<br>c={0.65, 0.75, 0.85, 0.95}<br>o=NO.	r={1,2,3}<br>q={0.05,0.06, 0.07, 0.08, 0.09, 0.1, 0.25, 0.35, 0.5}<br>c={0.65, 0.75, 0.85, 0.95}<br>o=100
QUBIC2	q={0.05,0.06, 0.07, 0.08, 0.09, 0.1, 0.25, 0.35, 0.5}<br>c={0.65, 0.75, 0.85, 0.95}<br>o=NO.	q={0.05,0.06, 0.07, 0.08, 0.09, 0.1, 0.25, 0.35, 0.5}<br>c={0.65, 0.75, 0.85, 0.95}<br>o=100
Unibic	div={ncol(input_matrix), max(floor(ncol(input_matrix)/3),1),<br>max(floor(ncol(input_matrix)/5),1), max(floor(ncol(input_matrix)/10),1),<br>max(floor(ncol(input_matrix)/15),1), max(floor(ncol(input_matrix)/20),1)}<br>q={0.1, 0.25, 0.35, 0.5}<br>t={0.65, 0.75, 0.85, 0.95}<br>nbic=NO.	div={ncol(input_matrix), max(floor(ncol(input_matrix)/3),1),<br>max(floor(ncol(input_matrix)/5),1), max(floor(ncol(input_matrix)/10),1),<br>max(floor(ncol(input_matrix)/15),1), max(floor(ncol(input_matrix)/20),1)}<br>q={0.1, 0.25, 0.35, 0.5}<br>t={0.65, 0.75, 0.85, 0.95}<br>nbic=100
EBIC	n=[1000,2000,3000,5000]<br>x=[0.5,0.6,0.7,0.85,0.9]<br>b=NO.	n=[1000,2000,3000,5000]<br>x=[0.5,0.6,0.7,0.85,0.9]<br>b=100
ISA2	no.seeds = {2,5,10, 20, ..., 100, 125, 150, 200}<br>thr.row* = {0.5, 1.0, 1.5, 2.0, 2.5, 3.0}<br>thr.column* = {0.5, 1.0, 1.5, 2.0, 2.5, 3.0, 3.5, 4.0, 4.5, 5.0}	no.seeds = {100, 200, 300, 500, 1000, 2000, 5000}<br>thr.row* = {0.5, 1.0, 1.5, 2.0, 2.5, 3.0}<br>thr.column* = {0.5, 1.0, 1.5, 2.0, 2.5, 3.0, 3.5, 4.0, 4.5, 5.0}
Spectral Biclustering	numberOfEigenvalues={1,2,3,4,5,6,7,8,9,11,13,15,18,20,25,30}<br>withinVar=NULL	numberOfEigenvalues={10, 20, 50, 100, 200, 300, 400, 500}<br>minr={10, 20, 50, 100, 200, 300, 400, 500}<br>withinVar=NULL
Fabia	pnum=NO.<br>thresZnum=c(0.05,0.2,0.35,0.5,0.65)<br>thresLnum=c(*,0.05,0.2,0.35,0.5,0.65)	pnum=seq(2,ncol(input_matrix),by=2)<br>thresZnum=c(0.05,0.2,0.35,0.5,0.65)<br>thresLnum=c(*,0.05,0.2,0.35,0.5,0.65)
RecBic	r={ncol(input_matrix), max(floor(ncol(input_matrix)/3),1),<br>max(floor(ncol(input_matrix)/5),1), max(floor(ncol(input_matrix)/10),1),<br>max(floor(ncol(input_matrix)/15),1), max(floor(ncol(input_matrix)/20),1)}<br>q={0.05,0.06, 0.07, 0.08, 0.09, 0.1, 0.25, 0.35, 0.5}<br>c={0.65, 0.75, 0.85, 0.95}<br>o=NO.<br>x=2	r={ncol(input_matrix), max(floor(ncol(input_matrix)/3),1),<br>max(floor(ncol(input_matrix)/5),1), max(floor(ncol(input_matrix)/10),1),<br>max(floor(ncol(input_matrix)/15),1), max(floor(ncol(input_matrix)/20),1)}<br>q={0.05,0.06, 0.07, 0.08, 0.09, 0.1, 0.25, 0.35, 0.5}<br>c={0.65, 0.75, 0.85, 0.95}<br>o=100<br>x=5
BiCoN	_	size={2000, 3000, 4000, 5000}<br>K={20, 30, 40, 50}<br>evaporation={0.3, 0.5, 0.7}<br>L_g_max={200, 500, 1000, 2000}<br>L_g_min=5
DESMOND	_	alpha={0.3, 0.5, 0.7}<br>q={0.05, 0.1, 0.15, 0.2}<br>p_val={0.05, 0.01, 0.001}<br>d={'UP', 'DOWN"}#integrate the results of "UP" and "DOWN";<br>ns=3 for the datasets of adi,liv,pan,coc which are of few conditions

mosbi	_	algo={qubic, unibic, qubic2}<br>row_threshold={0.001, 0.05, 0.1, 0.2, 0.5}<br>col_threshold={0.001, 0.05, 0.1, 0.2, 0.5}
RUBic	mnr={2, 5, 10, 20, 50}<br>mnc={2, 5, 10}<br>threshold={1, 1.5, 2.0, 2.5, 3.0}<br>num_bicluster=NO.	mnr={2, 5, 10, 20, 50}<br>mnc={2, 5, 10}<br>threshold={1, 1.5, 2.0, 2.5, 3.0}<br>num_bicluster=100
MESBC	K=NO.	K={floor(ncol(input_matrix)/10),<br>floor(ncol(input_matrix)/8),floor(ncol(input_matrix)/6),<br>floor(ncol(input_matrix)/4), floor(ncol(input_matrix)/2), ncol(input_matrix)}
Transbic	e0={0.1, 0.12, 0.15, 0.18, 0.2,0.22, 0.25}<br>mfra(γ0)={0.8, 0.82, 0.85, 0.88, 0.9}<br>tfra={0.75, 0.8, 0.85, 0.9}<br>cfra(σ)=0.75<br>sfra={0.3, 0.7}<br>tNum=10<br>(minC=1for identifying all expression patterns except constant-upregulated)	e0={0.15, 0.18, 0.2,0.22, 0.25}<br>mfra(γ0)={0.8, 0.82, 0.85, 0.88, 0.9}<br>cfra(σ)={0.5, 0.75, 0.9}<br>sfra={0.3, 0.5, 0.7, 0.9, 1}<br>tfra=0.9<br>minR=5

Table S1. The results of hyperparameter tuning on synthetic and real datasets. NO. is the number of implanted biclusters. For FABIA , its extractBic function automatically determines the most appropriate values for the parameter thresLnum marked by *.
